# Computational Screening of Amino-Functionalized Molecules for Direct Air Capture of CO_2_

**DOI:** 10.1021/acs.jpca.5c03392

**Published:** 2025-09-18

**Authors:** Chenhao Li, Sergio Vernuccio, Peyman Z. Moghadam

**Affiliations:** † School of Chemical, Materials and Biological Engineering, 7315 The University of Sheffield, Sheffield S1 3JD, U.K.; ‡ School of Chemistry and Chemical Engineering, University of Southampton, Southampton SO17 1BJ, U.K.; § Department of Chemical Engineering, University College London, London WC1E 7JE, U.K.

## Abstract

Direct air capture (DAC) of CO_2_ is a promising strategy for mitigating global carbon emissions by removing CO_2_ from the atmosphere. A critical factor in enhancing the efficiency of DAC is the design of functionalized materials with strong CO_2_ binding capabilities. This study screens a variety of amino-functionalized molecules, utilizing MP2 and density functional theory calculations, to identify promising candidates for CO_2_ capture under dry and humid conditions. The analysis determined the most stable configurations of CO_2_ and water with 15 amino-functionalized molecules. Amino acids such as arginine, 7-azaindole, 1,5,7-triazabicyclo-[4.4.0]­dec-5-ene, and melamine demonstrated the strongest CO_2_ binding energies, ranging from −17 to −19 kJ/mol. This is the result of both Lewis acid–base interactions between the electron-deficient carbon of CO_2_ and a N atom and hydrogen bonding. Generally, all of the amino groups exhibited a stronger binding affinity with water, attributed to the formation of stable hydrogen bonds between an electron-rich N atom and the hydrogen atoms of water. To guide the design of porous host structures incorporating these molecules as functional groups, the study was extended to hypothetical systems where multiple functional groups can essentially “sandwich” CO_2_, promoting simultaneous binding. In these scenarios, the repulsion between functional molecules emerged as a critical factor increasing the overall CO_2_ binding energy to ca. −30 to −40 kJ/mol. This analysis enabled the identification of optimal pore sizes for the design of functionalized frameworks to maximize the CO_2_ capture efficiency.

## Graphical abstract

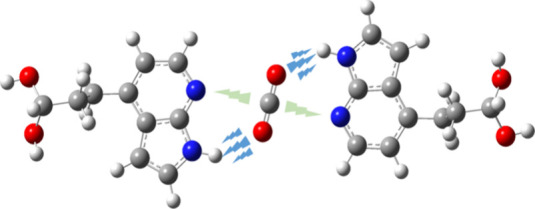

## 1 Introduction

Global warming, driven by the rise in the level of atmospheric carbon dioxide (CO_2_), has emerged as a global critical concern. In 2022, CO_2_ emissions related to the energy sector reached a new record high of over 36.8 gigatonnes (Gt), marking a significant increase compared to previous years.^1^ To meet the Intergovernmental Panel on Climate Change target of limiting the temperature increase to 2 °C, established during the 2015 COP21 conference in Paris, CO_2_ capture rates must improve significantly, reaching 10 Gt per year by 2050 and 25 Gt per year by the end of the century.^2^ In this context, CO_2_ capture, utilization, and storage have become one of the most critical and pressing challenges in today’s society.^3^ Direct air capture (DAC) technologies offer a promising solution for capturing CO_2_ directly from the atmosphere. These systems operate by using either liquid solvents or solid sorbents to bind with CO_2_ molecules, effectively separating them from ambient air.^4^ Compared to point-source CO_2_ capture, DAC benefits from processing cleaner ambient air, free of the impurities present in flue gas. This simplifies the capture process, potentially lowering associated costs, and provides greater flexibility in deployment locations. However, due to the low concentration of CO_2_ in ambient air (0.04 vol % in air, 400 ppm), DAC systems need to rely on adsorption or absorption technologies using materials with a strong binding affinity for CO_2_.^5^ Many DAC absorbents, including liquid amines and aqueous hydroxides, achieve high CO_2_ capture performances through chemisorption but require high temperatures (>120 °C) for regeneration.^6,7^ Solid adsorbent materials offer CO_2_ uptakes ranging from 0.1 to over 3 mmol/g and normally require milder regeneration conditions (ca. 100 °C).^8^ However, these materials may suffer from competitive adsorption of water under humid conditions. Currently, a wide range of sorbent families including polymers, carbons, zeolites, silicas, metal oxides, and metal–organic frameworks (MOFs) are heavily researched, in their unfunctionalized and functionalized forms, as viable solutions for DAC. Readers are encouraged to refer to important review papers for more details on DAC materials and technologies.^4,8–11^ Clearly, investing in new materials and DAC technologies is important as we urgently seek to mitigate climate change and transition to a low-carbon economy. In this context, MOFs have emerged as a promising class of adsorbents for DAC applications.^12–14^ MOFs are crystalline materials composed of inorganic nodes—either single metal atoms or metal clusters—connected by organic linkers, to form open networks with potential void spaces.^15^ Compared to traditional porous materials, MOFs offer tunability in characteristics such as surface area, pore size and shape, and surface chemistry. Additionally, they can be tailored to exhibit selective sorption properties through ligand and postsynthesis functionalization.^16–18^ Unmodified MOFs generally exhibit a low affinity and capacity for separating CO_2_ from air. To address this, various functionalized MOFs have been developed for selective CO_2_ capture, with N-containing molecules among the most commonly used groups.^19–22^ Functionalization with amino molecules is an effective strategy to increase the affinity of MOFs toward CO_2_, benefiting both adsorption strength and separation selectivity.^23–28^ For example, Chen et al.^29^ functionalized MOF-808 using a group of amino acid anions (AAs) and polyamine-substituted anion (PAs). Their experimental results demonstrated that the functionalized MOFs showed enhanced performance in the DAC of CO_2_, particularly under humid conditions. Two MOFs, namely, MOF-808-Lysine (Lys) and MOF-808-tris­(3-aminopropyl)­amine (TAPA), exhibited the highest CO_2_ uptakes at 400 ppm and 298 K in dry conditions, reaching 0.6 and 0.5 mmol/g, respectively. These CO_2_ uptake values were increased to 1.2 and 0.9 mmol/g when 50% relative humidity was introduced. Bose et al.^30^ tested *N*,*N*′-dimethylethylenediamine (mmen) appended Mg_2_(dobpdc) (dobpdc = 4,4′-dioxido-3,3′-biphenyldicarboxylate) MOF for DAC and obtained CO_2_ uptake of 1.7 mmol/g under 50% relative humidity at 296 K. Many metal nodes in MOF adsorbents are susceptible to hydrolysis by water, which can cleave metal–ligand bonds and chemically degrade the framework. Water inside MOF pores can both hinder the desired adsorption—through competitive binding, pore blocking, or hydrolysis—and, in some MOFs, enhance CO_2_ uptake.^31,32^ In this context, several reports show that the level of CO_2_ uptake increases under humid conditions for some MOFs, particularly amine-functionalized frameworks. In these cases, water creates new adsorption sites or alters the pore polarity to favor CO_2_ binding.^29,33–35^

Computational studies have also been carried out to investigate the interactions between CO_2_ and amino-functionalized molecules.^36,37^ For instance, Vogiatzis et al.^38^ reported a comprehensive computational analysis of CO_2_ interactions with N-containing organic heterocycles. They compared different DFT levels of theory, finding that Lewis acid–Lewis base interactions between the carbon of CO_2_ and the N of the heterocycle and hydrogen bonds play a significant role in the stabilized geometry of the molecules. Cai et al.^39^ calculated a CO_2_ binding energy of −9.7 kJ/mol with the −NH_2_ group of IRMOF-3. Zhu et al.^40^ developed a diamine-appended MOF called pip2-Mg_2_(dobpdc) (pip2 = 1-(2-aminoethyl)­piperidine) and used DFT to calculate the CO_2_ binding energy of −48.6 kJ/mol when the structure was loaded with 1.5 CO_2_ molecules per diamine at 30 °C. The predicted binding energy was within the experimental heat of adsorption value of −53 kJ/mol. Given the growing importance of designing functionalized MOFs for CO_2_ capture, it is imperative to develop a systematic computational approach to analyze the interactions between CO_2_ and the amino-functionalized molecules, which have shown a lot of promise for efficient carbon capture technologies. In this work, we computationally screened 15 amino-functionalized groups, which could potentially be incorporated into MOFs, and studied their binding energies with CO_2_ and water to evaluate their potential for DAC applications under dry and humid conditions. The thermal degradation of amines imposes a limitation on the maximum operating temperature during the desorption step, which, in turn, affects the overall energy performance of the CO_2_ capture process. Typically, primary and secondary amines degrade at temperatures of 100–130 °C. Tertiary amines tend to exhibit greater thermal stability. Cyclic amines are known to degrade by ring opening and closing at temperatures of 150–160 °C.^41^ Overall, in addition to high CO_2_ affinity and capacity, amino-functionalized solid sorbents must withstand thousands of low-pressure adsorption/desorption cycles in humid, oxygenated air without framework hydrolysis, pore collapse, amine decomposition, or leaching, all while remaining compatible with low-temperature regeneration to keep energy costs down.

More importantly, we developed hypothetical pore models to account for pore confinement effects, where CO_2_ molecules interact with multiple functional groups in the MOF. Our findings reveal several functionalized molecules with strong CO_2_-interaction energies that could be employed in the design of novel porous materials for DAC. DAC of CO_2_ has geographical dependencies because performance and costs are governed by local climatic conditions (e.g., temperature and humidity) and ambient CO_2_ concentration; consequently, geographic factors strongly influence sorbent selection and process configuration. In this work, we focus on the binding of CO_2_ with amino-functionalized molecules, in both the presence and absence of water, providing insights that are relevant across diverse environments and applications.

## 2 Computational Details

All density functional theory (DFT) calculations were conducted using the Gaussian 16 software package.^42^ Cluster models were employed to simulate and analyze the interactions of CO_2_ and water with various amino-functionalized molecules. The cluster models were constructed from appropriate functionalized materials, including MOFs, by replacing the metal nodes with hydroxyl capping groups.^43^ As an example, Figure S1 shows the construction of glycine from MOF-808-Gly. Geometry optimizations to describe the interaction of CO_2_ and water with a single functionalized molecule were conducted using the MP2 level of theory with the 6-311+G­(d,p) basis set.^44^ All atoms were allowed to relax during the optimization to identify the configurations with a minimum energy. Electronic density calculations were performed using the same level of theory and basis set. Interaction region indicator (IRI) calculations were conducted at the B3LYP functional level, using the same basis set, using Multiwfn software and visualized with VMD.^45^

CO_2_ and water with multiple functional groups were investigated to guide the design of porous frameworks for CO_2_ capture. To avoid unrealistic lateral interactions between the functionalized molecules, the hydroxyl capping groups were frozen during the optimization process, while all other atoms were allowed to fully relax. The M06-L exchange-correlation functional with the 6-311+G­(d,p) basis set was used for all calculations involving multiple functional groups. Basis set superposition error (BSSE) correction was applied to all of the aforementioned calculations to remove the overlap of basis set between different molecules. Frequency calculations were performed at the same level of theory as the geometry optimizations to estimate the adsorption enthalpies at 298 K.

## 3 Results and Discussion

### 3.1 CO_2_ and Water Interactions with Amino-Functionalized Molecules

The adsorption behavior of CO_2_ and water in a microporous material is governed by both the chemical characteristics of the material and its textural properties, such as the pore size and surface area. We first began our study by calculating the binding energies of CO_2_ and water with 15 different amino-functionalized molecules. To account for the presence of different adsorption sites, we evaluated several initial configurations of the adsorbate for each functionalized molecule. These initial configurations were selected based on possible hydrogen-bonding and electrostatic interactions with the functionalized molecules. Figure 1 illustrates three possible binding configurations of CO_2_ and tris­(2-aminoethyl)­amine (TAEA), as an exemplary functionalized molecule, with estimated binding energies and characteristic interatomic distances for each configuration.

**Figure 1:**
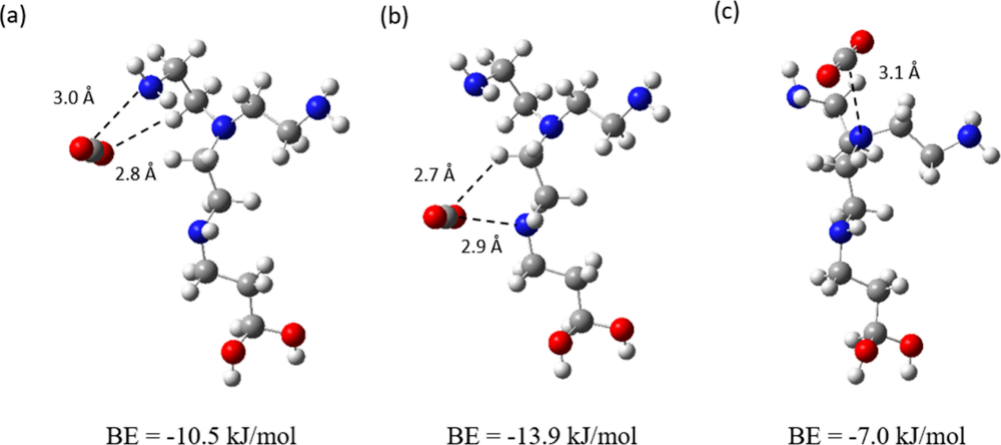
Different optimized configurations of CO_2_ interacting with a primary (a), secondary (b), and tertiary (c) amino functional group of an exemplary functionalized molecule (TAEA). Gray, white, red, and blue atoms correspond to C, H, O, and N, respectively. The dashed lines represent the distances in angstroms (Å) between pairs of atoms identified as primary interaction sites. The numbers below each configuration represent the CO_2_ binding energy.

The binding energy (BE) of CO_2_ and water for all possible configurations was calculated by using eq 1, where *E* indicates the electronic energy.

$$\mathrm{BE}=E_{\mathrm{FM}+\mathrm{adsorbate}}-E_{\mathrm{FM}}-E_{\mathrm{adsorbate}}+E_{\mathrm{BSSE}}$$ 1

Specifically, *E*_FM+adsorbate_ is the total energy of the functionalized molecule interacting with the adsorbate, *E*_FM_ is the energy of the functionalized molecule, *E*_adsorbate_ is the energy of the adsorbate (CO_2_ or water), and *E*_BSSE_ is the BSSE correction. Configurations with the most negative binding energies indicate the strongest interactions between the functionalized molecules and the adsorbate. The configurations in Figure 1 describe the interactions of CO_2_ with the primary (a), secondary (b), and tertiary (c) amino functional groups of TAEA. A significant contribution to the binding energy arises from the Lewis acid–base electrostatic interaction between the N of the amino group and the O of CO_2_. This is evidenced by the N–O­(CO_2_) interatomic distances, which are observed to range between 2.9 and 3.1 Å. Although the tertiary amino functional group is the strongest Lewis base of the molecule, the interaction of CO_2_ with the secondary (b) and primary (a) amino groups results in higher CO_2_ binding energies. This is due to the additional interaction between an electron-rich O atom of CO_2_ and a H atom of the functionalized molecule. Specifically, the configuration in Figure 1b exhibits a H–O­(CO_2_) distance of 2.7 Å, resulting in a binding energy of −13.9 kJ/mol.

Figure 2 presents the most stable MP2-calculated binding energies and configurations of CO_2_ for the 15 N-containing groups considered in this study. Amino acids such as arginine (Arg), 7-azaindole, 1,5,7-triazabicyclo-[4.4.0]­dec-5-ene (TBD), and melamine exhibit pronounced amphoteric properties, resulting in both (i) Lewis acid–base interactions between the electron-deficient carbon of CO_2_ and a N atom and (ii) hydrogen-bonding interactions. It can be observed that the groups that form two O­(CO_2_)–H bonds (melamine, TBD, etc.) show stronger binding with CO_2_ compared to those that primarily rely on vdW interactions (Gly, Lys, etc.) or those that generate single O­(CO_2_)–H bonds (TAEA, TAPA, etc.). These dual interactions result in the strongest CO_2_ binding for Arg (−17.0 kJ/mol), 7-azaindole, (−18.4 kJ/mol), TBD, (−17.5 kJ/mol), and melamine (−19.0 kJ/mol) among all of the functional groups studied. DFT calculations based on the M06-L functional result in CO_2_ binding energies ranging from −13 to −23 kJ/mol for the amino functional groups studied (Table S2).

**Figure 2:**
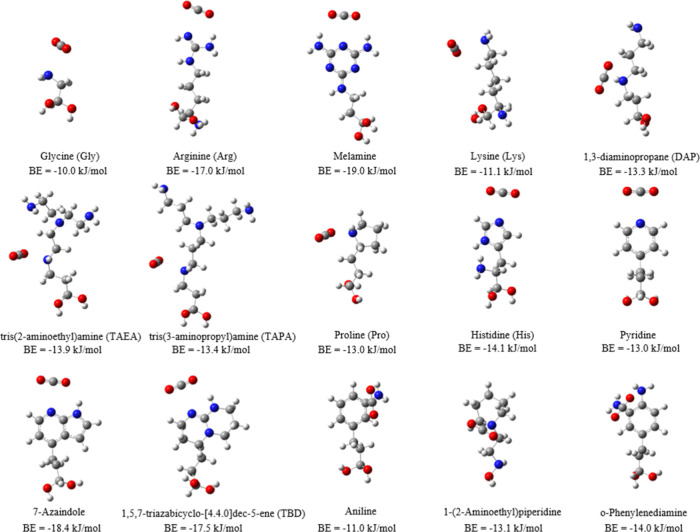
Most stable configurations of a CO_2_ molecule binding with amino-functionalized molecules. The numbers below each configuration represent the CO_2_ BE. Gray, white, red, and blue atoms correspond to C, H, O, and N, respectively.

Given the importance of competitive adsorption of CO_2_ and water in the design of adsorbent materials, we also computed the binding energies between water and the 15 functional molecules. Figure 3 presents the most stable configurations of a water molecule interacting with the functional molecules. The primary interaction between the functionalized molecules and water involves the formation of a hydrogen bond between an electron-rich N atom and the H­(H_2_O) atom. A secondary interaction involves vdW forces between the O atom and the NH or NH_2_ groups. The observed hydrogen-bonding distances range from 1.9 to 2.9 Å, while the angles of O–H–C­(N) and N–H–O span from 88° to 173°. These values are generally consistent with the established criteria for hydrogen bonding.^46^ The estimated electronic binding energies of CO_2_ and water with the investigated groups, along with their corresponding differences, are presented in Table 1. The negative value of BE_(H_2_O)_–BE_(CO_2_)_ in Table 1 shows a stronger binding affinity with water compared to that with CO_2_ for all of the functionalized molecules. Among all of the molecules studied, Arg exhibits the least negative value for BE_(H_2_O)_–BE_(CO_2_)_ (−5.6 kJ/mol), indicating its potential for CO_2_ capture under humid conditions. To mimic wet conditions and to provide some insight on the competitive binding of CO_2_ in the presence of H_2_O, we considered a scenario where we added a CO_2_ molecule to an optimized cluster of a preadsorbed water binding with an amino-functionalized molecule. Figure S2 shows the most stable configurations of CO_2_ binding with selected functional molecules: melamine, Arg, TBD, and 7-azaindole, in the presence of water. The results indicate that in the presence of water the binding of CO_2_ with the amino-functionalized groups is weakened due to the competitive adsorption. Under wet conditions, the CO_2_ binding energies for melamine, Arg, TBD, and 7-azaindole are −15.0, −10.6, −14.4, and −12.2 kJ/mol, respectively, which are weaker than the corresponding binding energies in the absence of water (−19.0, −17.0, −17.5, and −18.4 kJ/mol). In dry conditions, CO_2_ primarily interacts with the amino groups; however, when water is present, H_2_O has higher preferential interactions with the amino moieties compared to CO_2_, which binds simultaneously with water and the amino group. We note that in practice, CO_2_ capture in wet conditions will involve clustering of multiple water molecules around the primary adsorption sites (e.g., N-containing groups), together with confinement effects within the micropores, which are not fully captured by our quantum-chemical cluster approach.

**Figure 3:**
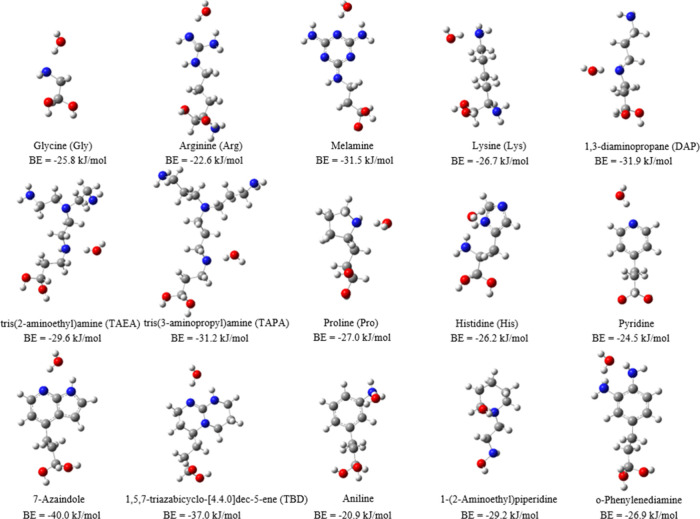
Most stable configurations of a water molecule binding with amino-functionalized molecules. The numbers below each configuration represent the H_2_O BE. Gray, white, red, and blue atoms correspond to C, H, O, and N, respectively.

**Table 1:** Binding Energies in kJ/mol Calculated for CO_2_ (BE_(CO_2_)_) and Water (BE_(H_2_O)_) with Different Amino-Functionalized Molecules^a^

functionalized molecules	*–*BE_(CO_2_)_	*–*BE_(H_2_O)_	BE_(H_2_O)_ – BE_(CO_2_)_
melamine	19.0	31.5	–12.5
7-azaindole	18.4	40.0	–21.6
TBD	17.5	37.0	–19.5
Arg	17.0	22.6	–5.6
His	14.1	26.2	–15.6
ο-phenylenediamine	14.0	26.9	–12.9
TAEA	13.9	29.6	–15.7
TAPA	13.4	31.2	–17.8
DAP	13.3	31.9	–18.6
1-(2-aminoethyl)piperidine	13.1	29.2	–16.1
Pro	13.0	27.0	–14.0
pyridine	13.0	24.5	–11.5
Lys	11.1	26.7	–15.6
aniline	11.0	20.9	–9.9
Gly	10.0	25.8	–15.8

a The last column represents the difference between the calculated binding energies of water and CO_2_. Negative values mean a stronger binding interaction with water.

To study and visualize the nature of the interactions between the adsorbates and the functionalized molecules, IRI calculations were additionally performed. Figure 4 shows the results obtained for the three exemplary molecules: Arg, Lys, and Gly. The isosurface maps display the van der Waals interactions among CO_2_, water, and the amino-functionalized molecules and show that hydrogen bonds predominantly influence the binding energy. The vdW interactions between the electron-rich O–H_2_O atom and the electron-deficient H atom (Figure 4a–c) are indicated by aquamarine surfaces as in the case of the interactions between the electron-deficient C and the electron-rich N (Figure 4d–f). The scattered map depicted in Figure S3 for Gly binding CO_2_ and water shows that both these interactions correspond to similar values of the sign­(λ_2_)­ρ function (approximately −0.014). The point at the bottom of each peak in Figure S3a,b corresponds to an IRI minimum, representing the interaction isosurface between the atoms shown in Figure S3c,d. A peak located on the negative *x*-axis (sign­(λ_2_)­ρ < 0) indicates strong binding and corresponds to a blue isosurface in the IRI analysis. Conversely, peaks located on the positive *x*-axis (sign­(λ_2_)­ρ > 0) represent the repulsion between atoms and are displayed as red isosurfaces. The peaks located close to the origin indicate weak vdW interactions and are represented as green isosurfaces. On the other hand, the hydrogen bonds between water and the amino functional groups are significantly stronger than those formed with CO_2_, resulting in a more negative binding energy between water and Arg, Lys, and Gly, as shown in Table 1.

**Figure 4:**
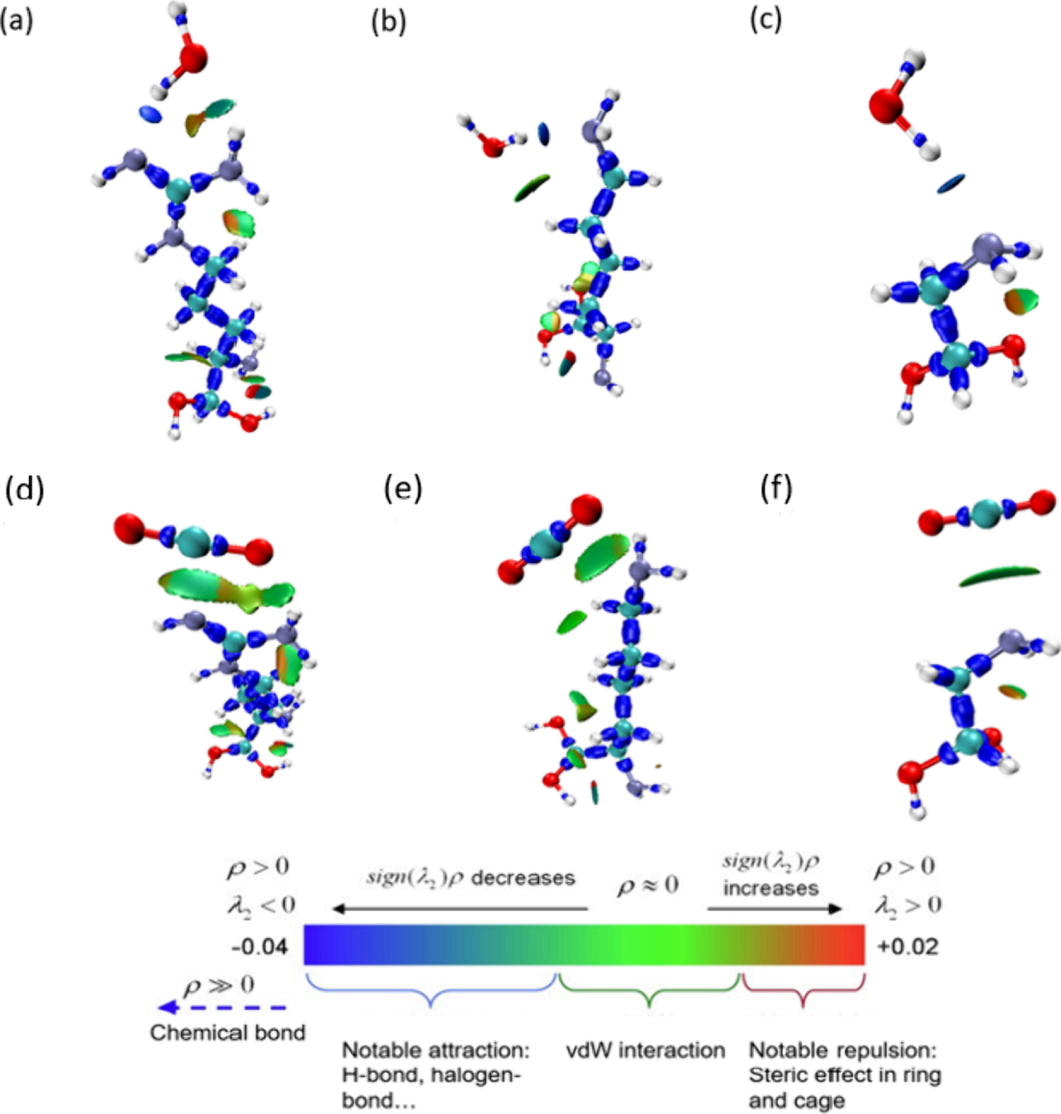
IRI calculations^47^ for water and CO_2_ binding Arg (a, d), Lys (b, e), and Gly (c, f) at IRI = 1.0. Red, turquoise, purple, and white atoms correspond to O, C, N, and H, respectively. ρ indicates the electron density, and sign­(λ_2_) denotes the sign of the second largest eigenvalue of the Hessian of ρ.

The enthalpy contribution is generally the primary driving force of the binding process, which is often accompanied by an entropy loss. Large and negative adsorption enthalpies reflect the intermolecular interactions between the adsorbent and the target adsorbate, including vdW forces and hydrogen bonds. The adsorption enthalpy (Δ*H*) was calculated by adding the zero-point (Δ*E*_ZPE_), rotational (Δ*E*_rot_), translational (Δ*E*_trans_), and vibrational (Δ*E*_vib_) contributions, as well as the pressure volume work Δ­(*PV*) to the previously calculated electronic BE, as shown in eq 2:

$$\Delta H=\mathrm{BE}+\Delta E_{\mathrm{ZPE}}+\Delta E_{\mathrm{rot}}+\Delta E_{\mathrm{trans}}+\Delta E_{\mathrm{vib}}+\Delta (\mathrm{PV})$$ 2

All of the terms in eq 2 were obtained from the electronic and frequency calculations previously described. It is worth noting that the calculated vibrational frequencies were positive for all of the cluster models, indicating thermodynamic stability of the species. The calculated Δ*H* values of water and CO_2_ binding with the amino-functionalized molecules are presented in Figure 5, where they are compared with the corresponding binding energies. These values are also tabulated in Table S1 for ease of reference. We note that the adsorption enthalpies for TAEA and TAPA are not included, as we were unable to achieve convergence in the frequency calculations due to computational limitations.

**Figure 5:**
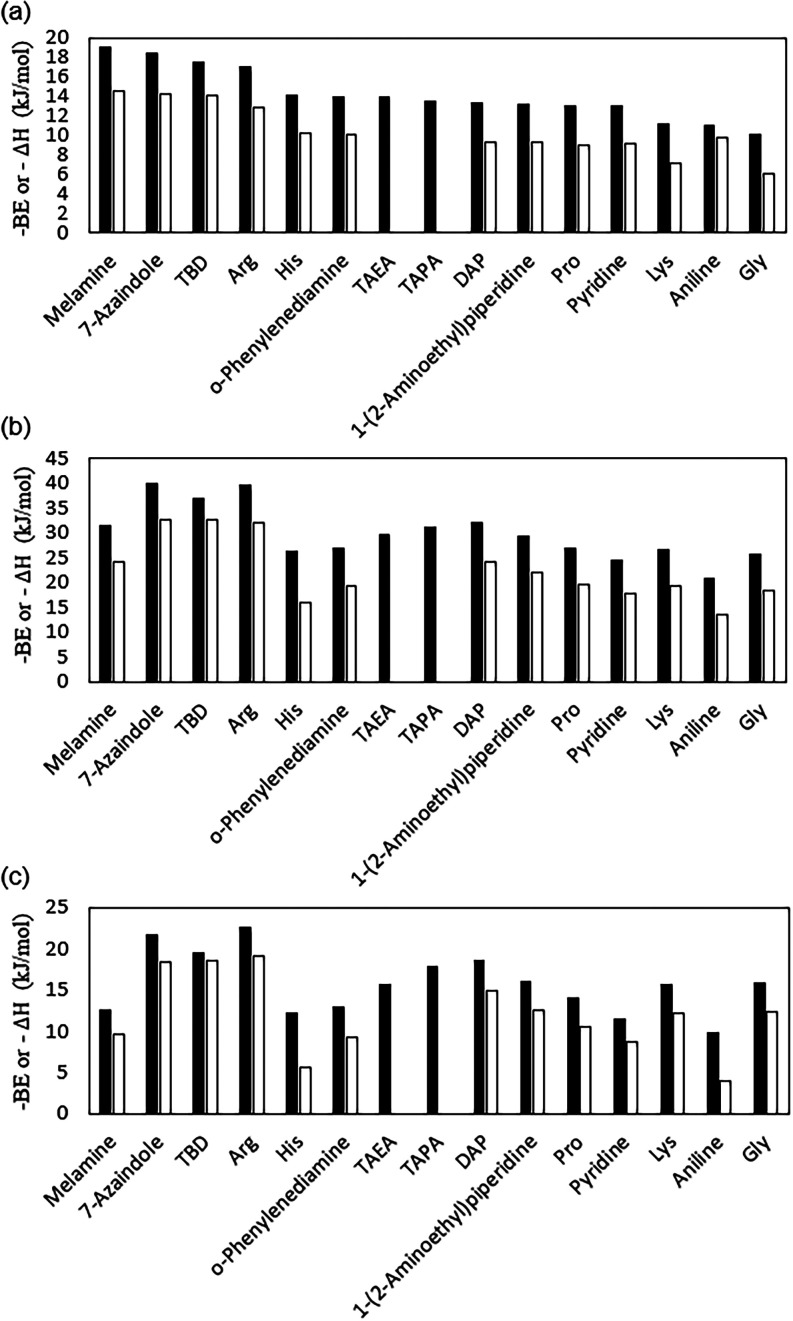
Comparison between calculated binding energies (black) and adsorption enthalpies at 298 K (white) for CO_2_ (a) and water (b). (c) Comparison between BE_(H_2_O)_–BE_(CO_2_)_ (black) and ΔH_(H_2_O)_–ΔH_(CO_2_)_ (white).

Figure 5a,b shows the comparison between the calculated electronic binding energy and the adsorption enthalpy. In all cases, the calculated Δ*H* values are less negative than the corresponding binding energy. However, the observed ranking of binding strength between the functional molecules and CO_2_ or water is generally maintained. Figure 5c shows a comparison between the differences of binding energies (BE_(H_2_O)_–BE_(CO_2_)_) and adsorption enthalpies (Δ*H*_(H_2_O)_–Δ*H*_(CO_2_)_) for water and CO_2_. These calculations indicate that melamine, Arg, 7-azaindole, and TBD exhibit the highest affinity for CO_2_ capture, with adsorption enthalpies ranging from −12.8 to −14.6 kJ/mol. None of the selected functionalized molecules preferentially adsorb CO_2_ over water when single-molecule interactions are considered. This analysis demonstrates that a screening based solely on the evaluation of the binding energies is generally sufficient to characterize functional groups’ affinity with CO_2_ and water, eliminating the need for additional and computationally intensive frequency calculations.

### 3.2 Applications of Functionalized Molecules to CO_2_ Capture

The identified top-performing functionalized molecules exhibit promising potential for CO_2_ capture, as evidenced by the strong CO_2_ binding energies predicted by DFT calculations. However, effective CO_2_ capture depends not only on the binding affinity but also on the pore environment, where CO_2_ molecules can simultaneously interact with multiple functional groups. To account for these multiple interactions, we extended the CO_2_ binding energy calculations to multifunctional-molecule systems, to inform the design of functionalized MOFs, that could incorporate these molecules as terminal groups. To do this, different DFT geometry optimization calculations were performed by varying the distance between the α-carbon of the diol group of two functional molecules with a single CO_2_ molecule sandwiched between the two. It is worth noting that the terminal −OH groups were frozen during the simulations to minimize the lateral interactions between the molecules and simulate the adsorption performance of a rigid structure. Figure 6 illustrates the results obtained for a 2-Arg system with the distance between the α-carbon atoms ranging between 19.0 and 33.0 Å. The red horizontal line represents the binding energy of CO_2_ with a single Arg molecule. Distances shorter than 20.6 Å result in a nonplanar structure, with the CO_2_ molecule moving out of the plane of the multi-Arg system, as shown in the inset for point I in Figure 6. The most negative binding energy (ca. −36 kJ/mol) was observed at a distance of 20.6 Å where CO_2_ is equidistant from the binding sites of the two Arg molecules (point II). At longer distances up to 23 Å (see points III and IV), the binding energy is significantly influenced by both functional molecules, as indicated by the progressive reduction in its value to ca. −21 kJ/mol. At greater distances, the binding energy of the multimolecule system converges toward the value observed for a single molecule (at the M06-L level of theory), indicating that CO_2_ interacts predominantly with one of the Arg molecules, while the influence of the second molecule becomes negligible (see point VI in Figure 6). Based on this analysis, a small mesopore size of approximately 20.0 Å is optimal when functionalizing rigid frameworks with Arg terminal groups to enhance the CO_2_ capture efficiency.

**Figure 6:**
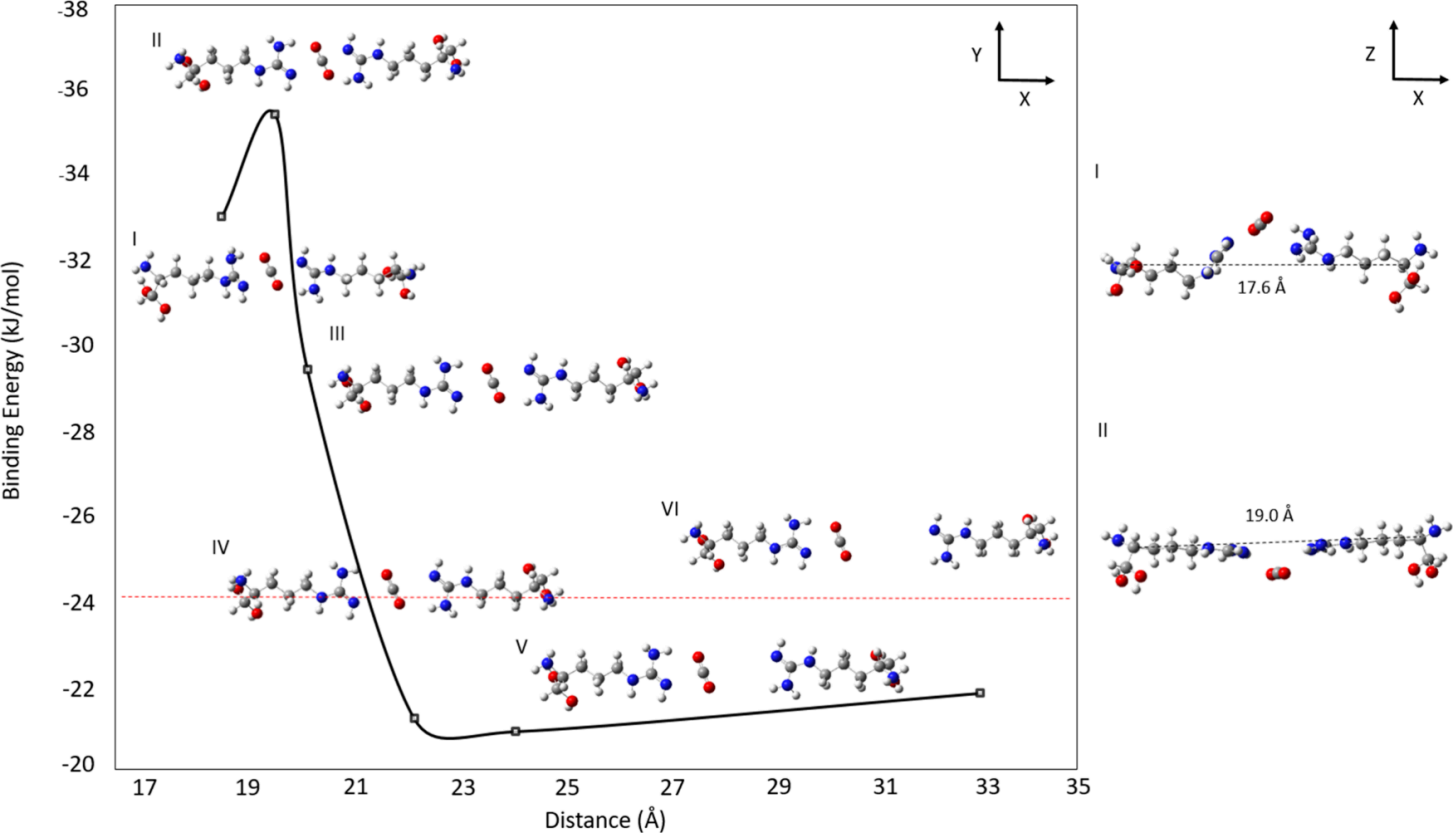
Binding energy between CO_2_ and two Arg molecules calculated at the M06-L level of theory with the 6-311+G­(d,p) basis set. The −OH terminal groups in Arg are kept frozen during the geometry optimization calculations. The *x*-axis indicates the distance between the two α-carbons of the diol groups. The horizontal red line represents the binding energy of CO_2_ and a single Arg molecule.

A similar approach was used to determine the optimal distance between the other functionalized molecules, resulting in the strongest CO_2_ binding interactions (Figures S4–S6). Figure 7 shows the two-molecule systems, namely, 7-azaindole, Arg, melamine, and TBD interacting with CO_2_. These molecules were identified as the top-performing groups from our previous CO_2_ binding energy analysis on the single-molecule systems (see Figure 2). The binding energy values shown in Figure 7 correspond to the DFT-relaxed structures obtained through the procedure depicted in Figure 6. All binding energy values exceed −30 kJ/mol, which is particularly advantageous for DAC applications where strong binding affinities are required. In particular, the 7-azaindole 2-molecule system results in the strongest CO_2_ binding energy (−38.5 kJ/mol), followed by Arg and TBD, each with binding energies of approximately −35 kJ/mol. In contrast to the trend observed in single-molecule simulations, the two-melamine system exhibits the longest N–C­(CO_2_) and H–O­(CO_2_) interatomic distances compared to other candidates and exhibits a relatively lower two-way binding energy of −32.7 kJ/mol. Among the other candidates, the two-TBD system shows the shortest N–C­(CO_2_) distance, while the two-Arg system displays the shortest H–O­(CO_2_) distance. However, the binding energy for these molecules remains lower than that of the two-7-azaindole conformation. This suggests that the interaction strength cannot be solely attributed to variations in the interatomic distances. To further investigate these interactions, we performed the IRI analysis (Figure S7) and generated a scatter map for all of the designed multimolecule systems for CO_2_ capture. Figure 8 reveals that while vdW interactions between C­(CO_2_) and the N atom in amino-functionalized molecules remain consistent across systems (sign­(λ_2_)­ρ ≈ 0.005), the hydrogen bond between O­(CO_2_) and H in the two-melamine system is relatively weaker (sign­(λ_2_)­ρ ≈ −0.011), which is consistent with our previous findings. Additionally, Figure 8 shows that melamine, Arg, and TBD all exhibit a clear peak at sign­(λ_2_)­ρ slightly greater than 0.012, indicating the repulsion between the O­(CO_2_) and N atom in amino-functionalized molecules. The combination of relatively strong H-bonding and weaker repulsion makes the two-7-azaindole system the most promising candidate for designing amino-functionalized rigid structures for CO_2_ capture.

**Figure 7:**
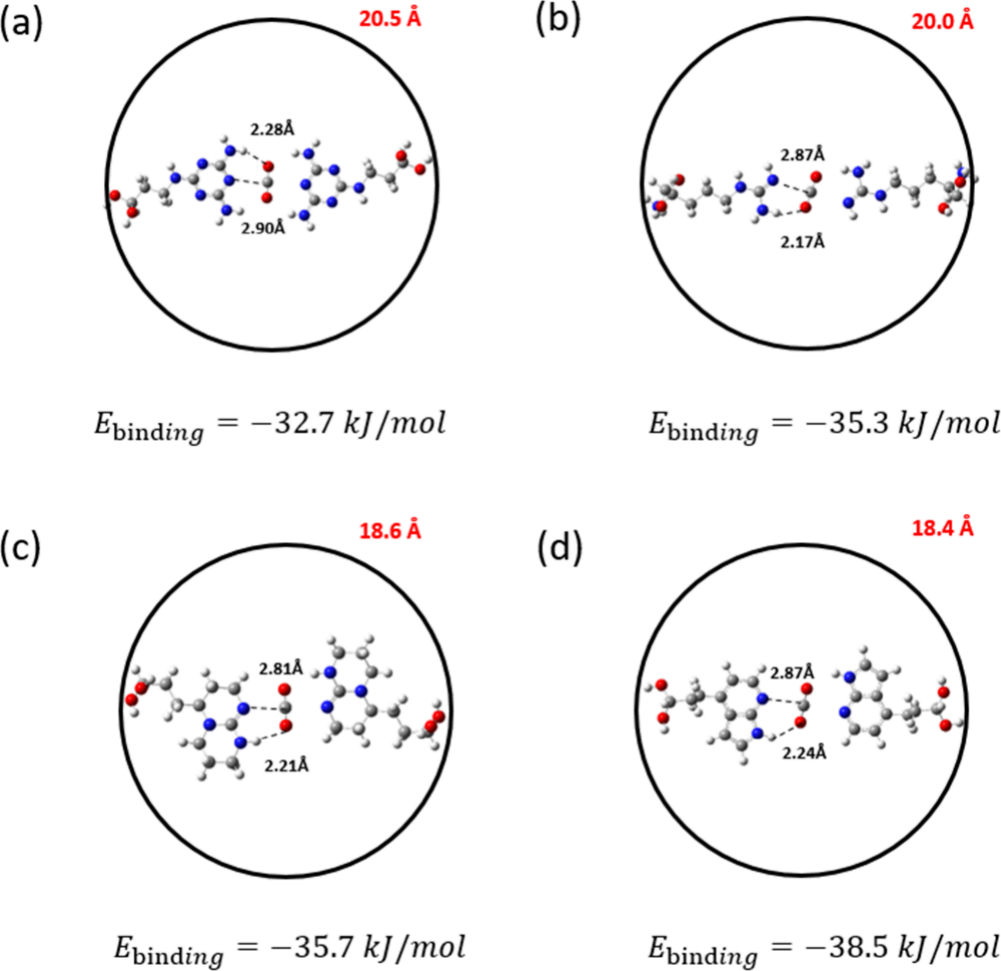
Most stable configurations of the hypothetical pore systems with two (a) melamine, (b) Arg, (c) TBD, and (d) 7-azaindole functionalized molecules for CO_2_ capture. Gray, white, red, and blue atoms correspond to C, H, O, and N, respectively. The terminal −OH groups were frozen during the simulations. The red numbers represent the distances in angstroms (Å) between the two diol α-carbon atoms of the functionalized molecules. The dashed lines represent the distances between the interacting atoms. The number below each configuration indicates the calculated CO_2_ binding energy.

**Figure 8:**
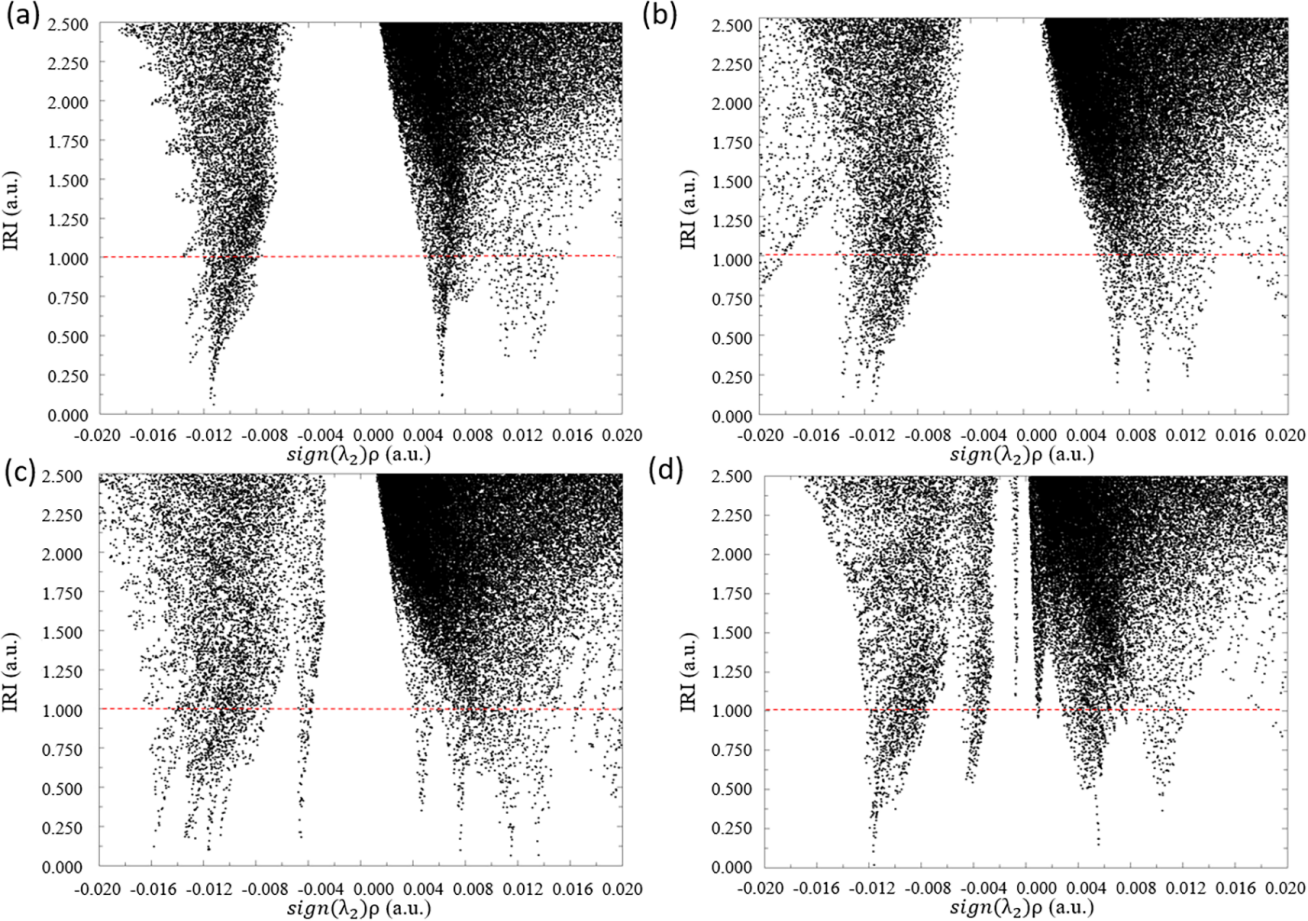
Scatter map of IRI vs sign­(λ_2_)­ρ of the designed system with two (a) melamine, (b) Arg, (c) TBD, and (d) 7-azaindole for CO_2_ capture. The points intersecting with the red dashed line (IRI = 1.0) correspond to the grid points constituting the isosurfaces shown in Figure S6.

An analogous study was conducted to investigate water binding with the two-molecule systems mentioned above (melamine, Arg, TBD, and 7-azaindole), as illustrated in Figure 9. In this case, the distance between the two diol α-carbon atoms was fixed to the value identified through the previous analysis in Figure 7 to assess the performance of a porous structure—specifically optimized for CO_2_ adsorption—in the presence of water. In all cases, water exhibits a more negative binding energy compared to that of CO_2_, indicating a preference of the investigated systems for water adsorption. During the geometry optimization calculations, the water molecules twisted to enable the hydrogen atoms of the amino groups to interact with the oxygen group in the ligand O­(H_2_O). This rearrangement of the functionalized molecules to accommodate water was not observed in the simulations involving CO_2_, where the molecules remained planar with the adsorbate.

**Figure 9:**
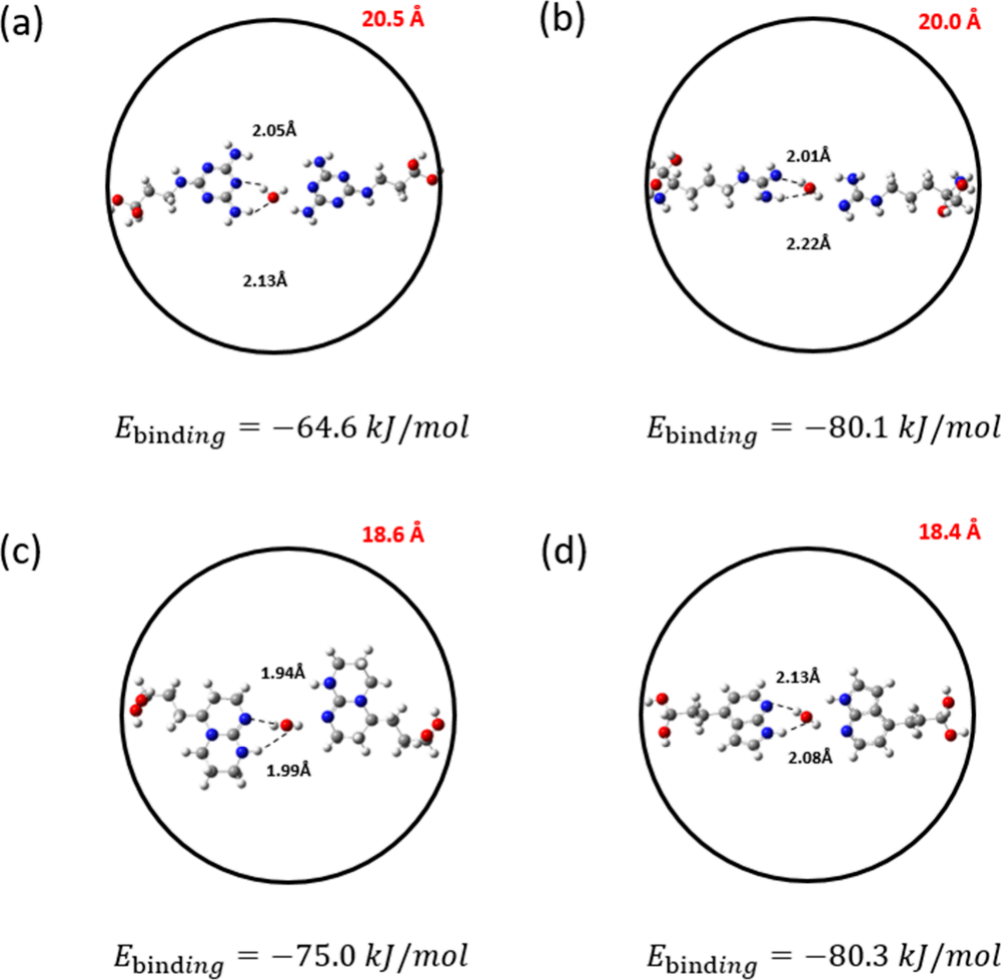
Most stable configurations of the hypothetical pore systems with two (a) melamine, (b) Arg, (c) TBD, and (d) 7-azaindole functionalized molecules for water capture. Gray, white, red, and blue atoms correspond to C, H, O, and N, respectively. The terminal −OH groups were frozen during the simulations. The red numbers represent the distances in angstroms (Å) between the two diol α-carbon atoms of the functionalized molecules. The dashed lines represent the distances between the interacting atoms. The numbers below each configuration represent the calculated H_2_O binding energy.

While all selected functionalized molecules exhibit strong interactions with water, melamine results in the weakest binding energy of −64.6 kJ/mol. This was attributed to the formation of relatively weak hydrogen bonds with distances of 2.05 and 2.13 Å for H­(H_2_O) and O­(H_2_O), respectively (Figure 9a).

## 4 Conclusions

In this study, we computationally screened different amino-functionalized molecules that can be incorporated into host materials such as MOFs for the DAC of CO_2_. Using MP2 and DFT calculations, we evaluated the binding energies and thermal enthalpies of adsorption for water and CO_2_ with 15 amino-functionalized molecules with the goal of identifying promising candidates for DAC under both dry and humid conditions. Our results show that 7-azaindole, TBD, melamine, and Arg interact strongly with CO_2_ with binding energy values ranging from −17.0 to −19.0 kJ/mol. Such relatively large binding energies are predominately influenced by the number and strength of hydrogen bonds formed between the atoms of the O­(CO_2_) and H from the functional molecules. These groups demonstrate greater potential for CO_2_ DAC compared to those that primarily rely on vdW interactions, e.g., Gly and Lys. To inform the design of rigid porous structures that incorporate amino-functionalized molecules as terminal groups, we generated hypothetical pore structures that enabled the simultaneous binding of CO_2_ with two functional groups. We found that the two-way interactions with CO_2_ result in significantly stronger binding energies of ca. −33 to −39 kJ/mol. In particular, CO_2_ strongly coordinates with the two molecules of 7-azaindole, resulting in a binding energy of −38.5 kJ/mol. Overall, the binding energy in a two-molecule system is influenced not only by the strength of the hydrogen bonds but also by the repulsive interactions between the functional molecules, which play a critical role in determining the overall binding energy. This means that multiamino-functionalized molecules such as 7-azaindole, TBD, melamine, and Arg could serve as multiple binding sites when CO_2_ is “sandwiched” between these groups inside a pore. We note that these results serve primarily as a prescreening tool for the identification of promising functional groups for MOF linkers. The ultimate optimization of MOF sorbents for DAC in humid environments may require further tuning of structural properties including pore size/shape, pore volume, surface area, and topology along with the binding properties of the amino-functionalized groups. The findings from this study offer valuable insights for the development of promising amino-functionalized materials for the efficient DAC of CO_2_.

## Supplementary Material



## Notes

Calculated adsorption enthalpies for CO_2_ and water with different amino-functionalized molecules; construction of an exemplary functional molecule cluster model (Gly) from MOF-808-Gly; IRI analysis of Gly; CO_2_ and water binding energies of single-molecule systems from M06-L calculations; binding energy of CO_2_ and two-melamine, 7-azaindole, and TBD molecules; IRI calculations for CO_2_ binding on two-melamine, Arg, TBD, and 7-azaindole; and coordination of all mentioned models (PDF)
