# Supplementary material for: Computational Screening of Amino-Functionalized Molecules for Direct Air Capture of CO2
Source: J Phys Chem A. 2025 Sep 18;129(39):9041–51. doi: 10.1021/acs.jpca.5c03392 (PMC12498499; doi:10.1021/acs.jpca.5c03392)
Supplement: Supplementary file 1 [file jp5c03392_si_001.pdf]

Supporting Information

Computational Screening of Amino-Functionalized Molecules for Direct Air Capture of CO_2

Chenhao Li†, Sergio Vernuccio†‡, Peyman Z. Moghadam⁂**

† School of Chemical, Materials and Biological Engineering, The University of Sheffield, Sheffield, S1 3JD, United Kingdom.

‡ School of Chemistry and Chemical Engineering, University of Southampton, Southampton, SO17 1BJ, United Kingdom.

⁂ Department of Chemical Engineering, University College London, London, WC1E 7JE, United Kingdom.

*Corresponding authors: Sergio Vernuccio (s.vernuccio@soton.ac.uk), Peyman Z. Moghadam (p.moghadam@ucl.ac.uk)

Table S1. Calculated adsorption enthalpies (in kJ/mol) at 298 K for CO_2 ($\Delta H_{(CO_2)}$) and water ($\Delta H_{(H_2O)}$) for different amino-functionalized molecules. The last column represents the difference between the calculated binding energies of water and CO_2. Negative values indicate that the binding of water is enthalpically more favourable than CO_2.

Functional groups	$-\Delta H_{(CO_2)}$	$-\Delta H_{(H_2O)}$	$\Delta H_{(H_2O)} - \Delta H_{(CO_2)}$
Melamine	14.6	24.3	-9.7
7-Azaindole	14.2	32.5	-18.3
TBD	14.1	32.6	-18.5
Arg	12.8	32.0	-19.2
o-Phenylenediamine	10.1	19.4	-9.3
TAEA			
TAPA			
DAP	9.3	24.2	-14.9
1-(2-Aminoethyl)piperidine	9.3	21.9	-12.6
Pro	9.0	19.5	-10.5
Pyridine	9.2	17.9	-8.7
Lys	7.2	19.3	-12.1
Aniline	9.7	13.7	-4.0
His	10.2	15.9	-5.7
Gly	6.0	18.3	-12.3

Table S2. Binding energies in kJ/mol calculated for CO_2 ($BE_{(CO_2)}$) and water ($BE_{(H_2O)}$) with different amino-functionalized molecules using M06-L functional and the 6-311+G (d,p) basis set.

Functional groups	$-\boldsymbol{BE}_{(\boldsymbol{CO_2})}$	$-\boldsymbol{BE}_{(\boldsymbol{H_2O})}$
Melamine	18.6	41.3
7-Azaindole	21.2	46.3
TBD	22.7	46.4
Arg	22.3	49.9
o-Phenylenediamine	16.8	32.2
TAEA	16.5	48.1
TAPA	18.0	35.0
DAP	19.0	37.2
1-(2-Aminoethyl)piperidine	20.1	33.7
Pro	17.3	31.6
Pyridine	15.7	27.1
Lys	15.5	31.5
Aniline	13.7	22.0
His	16.1	31.5

Gly	13.4	29.3

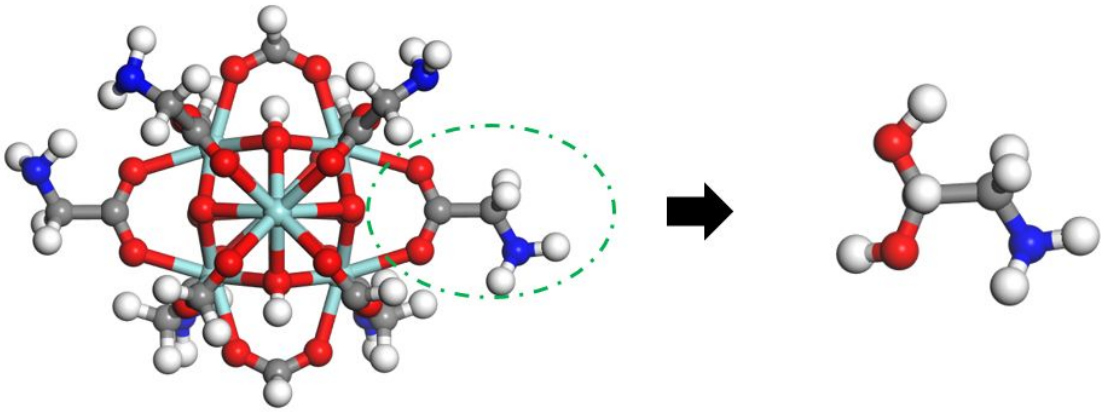

Figure S1. Construction of an exemplary functional molecule cluster model (Gly) from MOF-808-Gly. Grey, white, red, turquoise and blue atoms correspond to C, H, O, Zr and N.

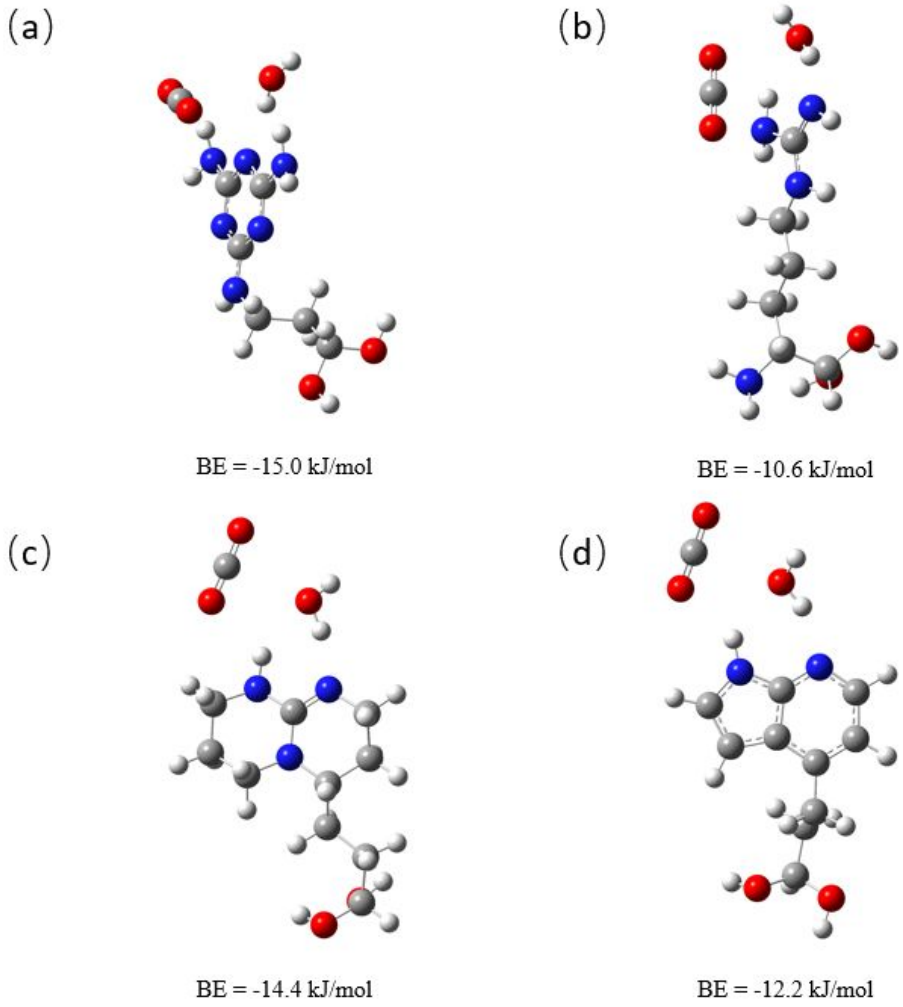

Figure S2. Most stable configuration of a CO_2 molecule binding with water molecules and **(a)** Melamine, **(b)** Arg, **(c)** TBD, **(d)** 7-Azaindole. Grey, white, red and blue atoms correspond to C, H, O and N, respectively. The number below each configuration represent the calculated CO_2 binding energy in the presence of water.

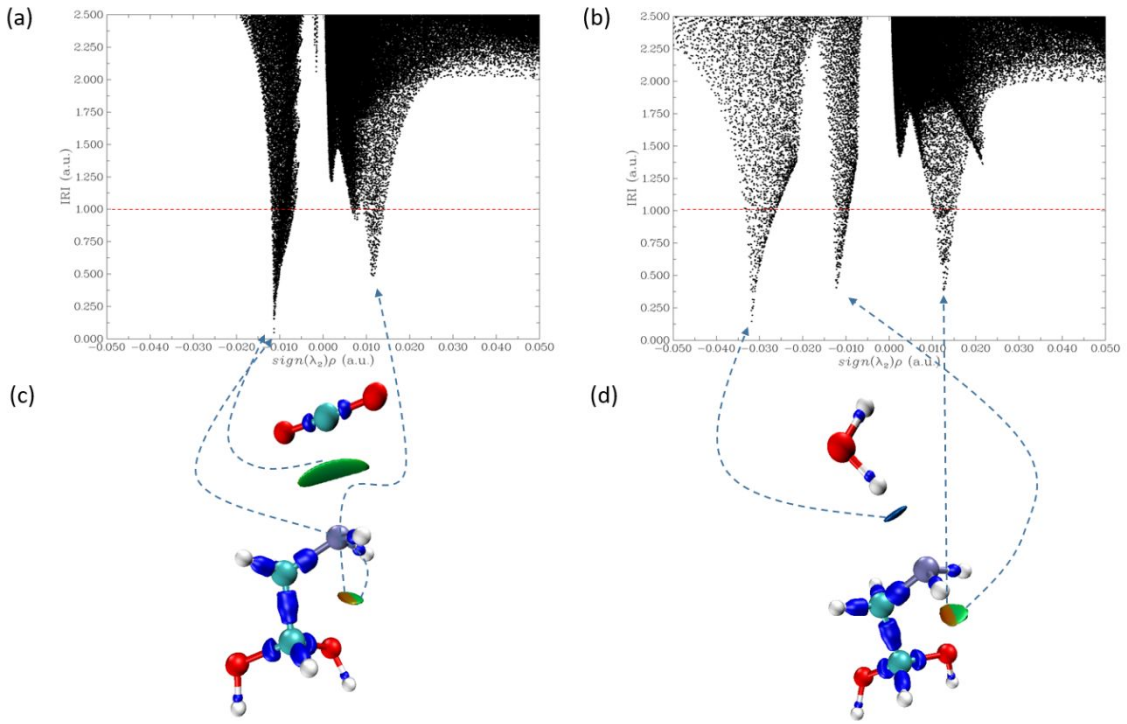

Figure S3. Interaction region indicator (IRI) analysis of Gly. Scatter map of IRI vs sign(λ_2)ρ for CO_2 **(a)** and water **(b)** binding with Gly. Isosurface map at IRI = 1.0 for CO_2 (c) and water (d) binding. Red, turquoise, purple, white atoms correspond to O, C, N and H, respectively. The arrows show the correspondence between IRI isosurfaces and the observed IRI minima.

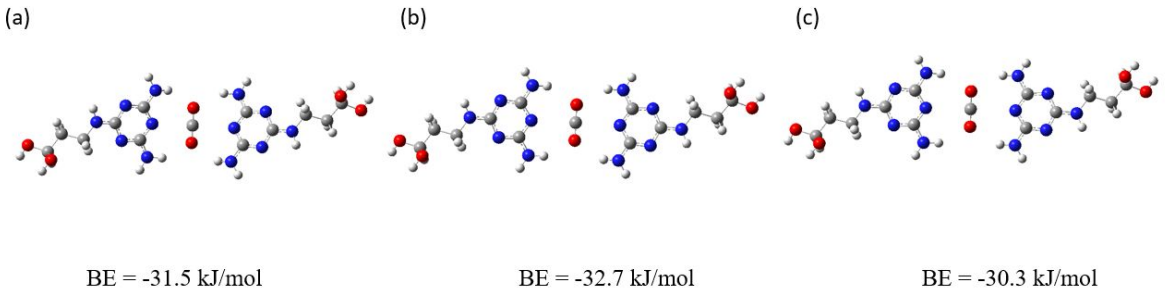

Figure S4. Binding energy of CO_2 and two Melamine molecules calculated at the M06L level of theory with the 6-311+G (d,p) basis set by freezing the -OH terminal groups. The distance between two dial alpha carbons from both Melamine molecules is **(a)** 19.8 Å, **(b)** 20.5 Å, **(c)** 20.8 Å. Grey, white, red and blue atoms correspond to C, H, O and N.

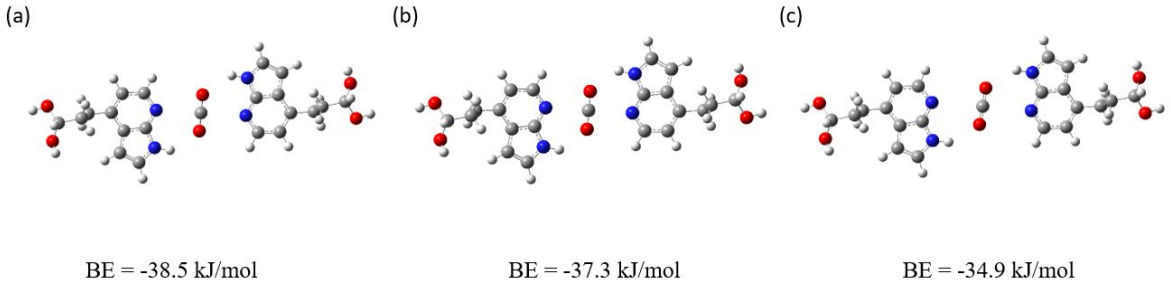

Figure S5. Binding energy of CO_2 and two 7-Azaindole molecules calculated at the M06L level of theory with the 6-311+G (d,p) basis set by freezing the -OH terminal groups. The distance between two dial alpha carbon from both Melamine molecules is **(a)** 18.4 Å, **(b)** 17.6 Å, **(c)** 19.1 Å. Grey, white, red and blue atoms correspond to C, H, O and N.

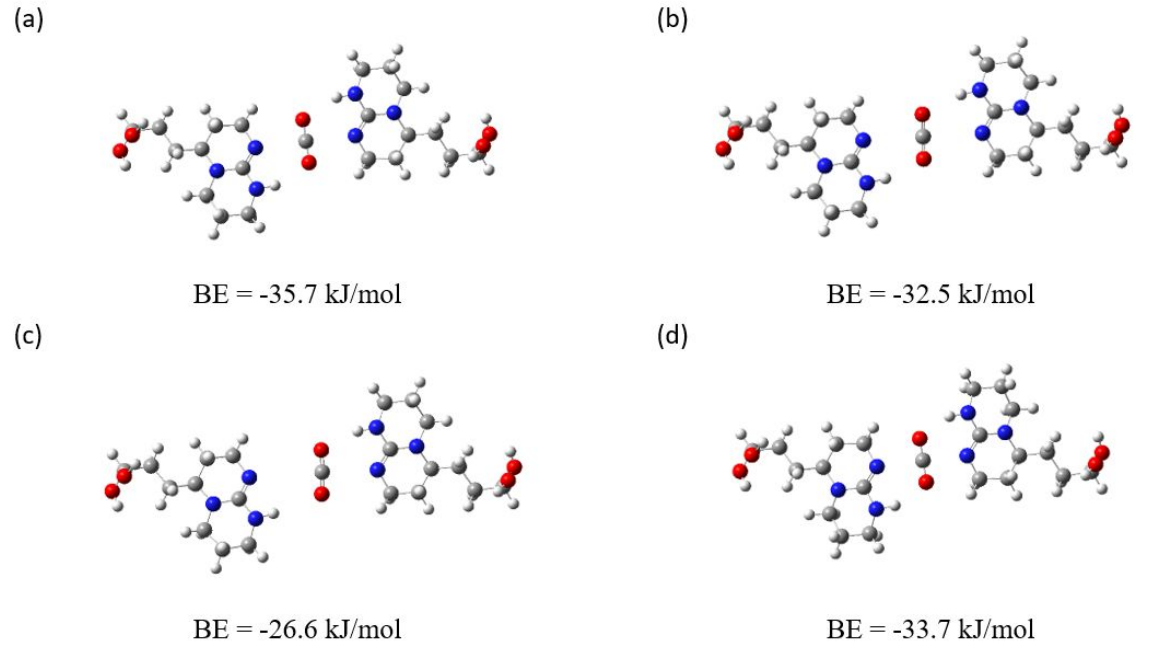

Figure S6. Binding energy of CO_2 and two TBD molecules calculated at the M06L level of theory with the 6-311+G (d,p) basis set by freezing the -OH terminal groups. The distance between two dial alpha carbon from both Melamine molecules is **(a)** 18.6 Å, **(b)** 19.5 Å, **(c)** 20.0 Å, **(d)** 18.0 Å. Grey, white, red and blue atoms correspond to C, H, O and N.

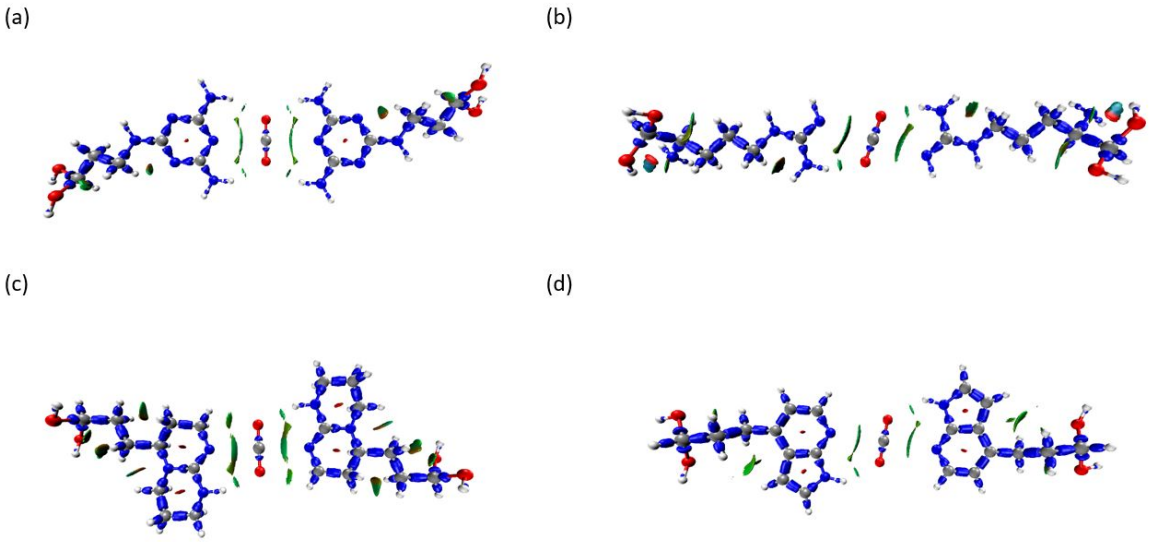

Figure S7. Interaction region indicator (IRI) calculations for CO_2 binding on two- **(a)** Melamine, **(b)** Arg, **(c)** TBD, **(d)** 7-Azaindole at IRI = 1.0. Red, turquoise, purple, white atoms correspond to O, C, N and H.

Geometry optimized coordinates:

Single-molecule calculations with CO_2

AA-Gly

C	2.06166200	-0.11092800	-0.29991500
O	2.41265100	-0.65096000	0.96171500
O	2.71959200	1.10805800	-0.54709900
H	3.65176400	0.98487800	-0.34389300
C	0.57193700	0.18982000	-0.28030900
H	0.31001300	0.70276300	-1.20997800
H	0.39500900	0.88299100	0.54757300
N	-0.28696000	-0.97604600	-0.11125900
H	0.00500800	-1.48911700	0.71564200
H	-0.20091200	-1.60636700	-0.90345900

75	H	3.08875000	-1.32021300	0.82892700
76	H	2.30023900	-0.84498600	-1.08535500
77	C	-2.90923200	0.23433800	0.10795500
78	O	-3.36488900	-0.41039000	-0.75587300
79	O	-2.50327300	0.90866600	0.97412900
80	AA-Arg			
81	C	-4.40797500	0.91903200	0.13540100
82	O	-3.45341800	1.93800200	0.04589100
83	O	-4.67478900	0.67004200	1.49554200
84	H	-5.06411300	-0.21819700	1.50834100
85	C	-3.86742600	-0.33704300	-0.55677400
86	H	-3.77514000	2.65983600	0.59701400
87	H	-5.34174500	1.22881600	-0.36527600
88	C	-2.52328000	-0.79159400	0.02136700
89	C	-1.31602000	-0.14823000	-0.66233300
90	C	-0.00856700	-0.59188200	-0.01601800
91	N	1.11879300	0.00982700	-0.71786300
92	H	-2.50850000	-0.58157600	1.09649100
93	H	-1.40456000	0.94061400	-0.60202100
94	H	0.08491300	-1.68098300	-0.08444400
95	C	2.44264800	-0.25417300	-0.38593900
96	N	-4.88701800	-1.37136400	-0.31235900

97	H	-4.53499600	-2.28550100	-0.57910000
98	H	-5.71641800	-1.19897800	-0.87626700
99	H	-3.74420000	-0.10484000	-1.62546800
100	H	-2.44840100	-1.88200000	-0.09292700
101	H	-1.29409300	-0.42612400	-1.72335100
102	H	-0.02640600	-0.31580000	1.05135600
103	H	0.96514100	0.97204400	-0.99043000
104	N	2.63211700	-1.39576400	0.38663000
105	N	3.47482300	0.39370200	-0.81806200
106	H	3.18198500	1.12665700	-1.46371200
107	H	3.59690400	-1.46687400	0.68676100
108	H	1.98441300	-1.51664200	1.15313900
109	C	6.01132200	0.34946700	0.39450500
110	O	6.38649200	1.27455200	-0.21133900
111	O	5.70296700	-0.57993600	1.03993400
112	Melamine			
113	C	-1.95000900	-1.28149200	0.10236800
114	N	-0.66329200	-1.64316400	0.09384500
115	C	0.18930200	-0.60687100	-0.03423900
116	N	-0.14521400	0.68885200	-0.14353000
117	C	-1.47125300	0.90056500	-0.14537100
118	N	-2.43129500	-0.02937900	-0.02453500

119	N	-2.86983700	-2.27159900	0.31422400
120	N	1.51093400	-0.92364500	-0.08828300
121	N	-1.88129100	2.19085900	-0.33879700
122	H	-2.53990900	-3.20947100	0.14284500
123	H	-3.81884400	-2.06021100	0.04290400
124	H	1.71523900	-1.86929100	0.20804800
125	H	-1.18129000	2.89536100	-0.16151700
126	H	-2.82877700	2.40186500	-0.06152500
127	C	2.55644900	0.07855600	0.06134500
128	C	3.90653700	-0.55473100	-0.25225600
129	C	5.03756300	0.44955800	-0.20456900
130	H	2.34321200	0.89180500	-0.63805000
131	H	2.55861900	0.50383100	1.07113000
132	H	3.88461800	-0.99834800	-1.25215300
133	H	4.14389300	-1.34641500	0.46865600
134	O	5.03557200	1.01706800	1.08741300
135	H	4.88810800	1.23702600	-0.95874300
136	O	6.24006600	-0.24210900	-0.47934600
137	H	5.77068400	1.63577700	1.14204800
138	H	6.84548200	0.36482900	-0.91369500
139	C	-5.26334700	0.60254100	0.00850200
140	O	-5.54575900	-0.51863000	-0.17080200

141	O	-5.03644400	1.73629700	0.18834800
142	AA-Lys			
143	C	3.14540100	0.78990200	0.50514700
144	O	2.18004100	1.19156500	1.43399900
145	O	3.15657000	1.70937700	-0.56200500
146	H	3.56512600	1.22346400	-1.29530900
147	C	2.80985400	-0.62120700	0.01021000
148	H	2.33673500	2.12760000	1.59963800
149	H	4.14352100	0.77530300	0.97740300
150	C	1.40034700	-0.71822600	-0.58246700
151	C	0.31901600	-1.06166900	0.44419500
152	C	-1.07152100	-1.11033100	-0.18733300
153	C	-2.15963700	-1.47914200	0.81424200
154	H	1.15558000	0.22478100	-1.08358000
155	H	0.33256400	-0.31729300	1.24524900
156	H	-1.07820000	-1.83967000	-1.01081200
157	N	-3.48012800	-1.44402000	0.16891500
158	H	-2.16643800	-0.74398100	1.62606100
159	N	3.81070300	-0.90163700	-1.03480600
160	H	3.55579300	-1.73710800	-1.55206800
161	H	4.72403300	-1.07645500	-0.62148000
162	H	2.89062300	-1.30492700	0.86899200

163	H	1.40386900	-1.49776000	-1.35778700
164	H	0.54957000	-2.03561900	0.89861600
165	H	-1.30696000	-0.13475200	-0.62923200
166	H	-4.19730300	-1.74714700	0.82257600
167	H	-3.49803800	-2.10392100	-0.60458600
168	H	-1.91830600	-2.45855700	1.25627500
169	C	-3.73487100	1.39037400	-0.36477500
170	O	-3.99976500	1.27138700	-1.49800200
171	O	-3.46906200	1.56860100	0.76175200
172	PA-DAP			
173	C	1.21987100	-0.84507900	-0.17443800
174	C	2.68589800	-0.45608200	-0.32705400
175	H	0.93807800	-0.77297900	0.88242200
176	H	1.07947100	-1.89908200	-0.47806300
177	H	2.80298900	0.59591700	-0.04134600
178	H	2.97941300	-0.54478000	-1.38317500
179	N	0.36292100	0.05937200	-0.94497500
180	H	0.74973900	0.12450900	-1.88504700
181	C	3.61752400	-1.32528300	0.52248900
182	N	5.04421400	-1.01010900	0.42376400
183	H	3.32957400	-1.23770200	1.57626300
184	C	-1.71400600	-0.42858100	0.29973600

185	C	-1.02157300	-0.41446000	-1.06102300
186	H	-1.48876500	0.49526200	0.84061100
187	H	-1.37756000	-1.27073400	0.91322600
188	H	-1.54872300	0.27992200	-1.72651200
189	H	-1.08339900	-1.41841800	-1.50816300
190	C	-3.21670000	-0.53958200	0.17301100
191	O	-3.76000700	-0.60157700	1.47739900
192	O	-3.48539100	-1.71042300	-0.57044100
193	H	-4.43990200	-1.79232300	-0.66179400
194	H	5.20381100	-0.04296000	0.69080500
195	H	5.35449600	-1.10093100	-0.53929900
196	H	3.48871800	-2.37830400	0.24712700
197	H	-4.64058300	-0.21705400	1.45647200
198	H	-3.62529700	0.33691000	-0.35250900
199	C	0.13865900	2.70569800	0.07024500
200	O	-0.82134900	2.87678600	-0.57884500
201	O	1.08799100	2.59997700	0.74684500
202	PA-TAEA			
203	C	0.13515200	-0.14132900	0.27622200
204	C	-1.30539600	0.15000300	-0.14033800
205	H	0.29836200	-1.22914400	0.28800500
206	H	0.27850700	0.21865700	1.30281900

207	H	-1.42754500	-0.08249900	-1.21309300
208	H	-1.48929500	1.22143100	-0.02980100
209	N	1.05700000	0.59173200	-0.58868000
210	H	1.01323700	0.21359700	-1.53320500
211	N	-2.26652700	-0.60840100	0.66654300
212	C	-3.21862000	0.21586100	1.41445400
213	C	3.08212800	-0.79980600	0.02916600
214	C	2.44696800	0.58557600	-0.13013300
215	H	3.07429600	-1.33529000	-0.92844200
216	H	2.52811000	-1.39986900	0.75657800
217	H	3.03801700	1.17388400	-0.83791900
218	H	2.47157900	1.11194200	0.83405300
219	C	4.51862600	-0.72777900	0.49808600
220	O	5.00443000	-2.05153300	0.61589400
221	O	5.23504100	0.01796300	-0.46440300
222	H	6.14828700	0.08859700	-0.16964300
223	N	-3.98659900	1.66565300	-0.44885900
224	C	-4.41428300	0.77291400	0.63570400
225	H	-4.79744800	2.12334100	-0.85634200
226	H	-3.56231300	1.12654300	-1.19870300
227	H	-5.04571700	-0.06047300	0.29115200
228	H	-5.01634400	1.36148400	1.33782300

229	C	-1.91547400	-2.81775600	-0.40455500
230	C	-2.89903900	-1.70503800	-0.05255600
231	N	-2.63847600	-3.89972000	-1.08137100
232	H	-1.39618900	-3.12385000	0.51528300
233	H	-1.16057300	-2.43733100	-1.10066700
234	H	-3.67730800	-2.12702100	0.59812500
235	H	-3.39570500	-1.38186700	-0.98664900
236	H	-3.24959600	-4.37257900	-0.42165600
237	H	-1.98871600	-4.59791500	-1.42893300
238	H	-2.65983900	1.04814400	1.85581100
239	H	-3.61076900	-0.39383900	2.24029400
240	H	5.70732800	-2.05827400	1.27163500
241	H	4.58202300	-0.22748300	1.47674100
242	C	0.35292000	3.33983000	-0.27169500
243	O	0.29802400	3.13939400	0.88167500
244	O	0.39774700	3.60169100	-1.41102100
245	PA-TAPA			
246	C	1.38002700	0.27424200	-0.20451500
247	C	-0.03282200	0.01766700	0.30769600
248	H	1.54503700	1.36250300	-0.28106200
249	H	1.48472700	-0.14194500	-1.21548300
250	H	-0.13310900	0.38367600	1.33727100

251	H	-0.20466000	-1.06247800	0.33016600
252	N	2.36063900	-0.40217000	0.64256900
253	H	2.31391900	-0.02124400	1.58569300
254	C	-1.08828100	0.70445800	-0.55432400
255	N	-2.44912500	0.33450500	-0.15472400
256	C	4.31279100	1.08804200	0.00110400
257	C	3.73931000	-0.32457800	0.15591300
258	H	4.29556500	1.61307900	0.96417000
259	H	3.72668900	1.67270300	-0.71336600
260	H	4.36948300	-0.89471800	0.84432000
261	H	3.76931200	-0.83783800	-0.81589800
262	C	5.74534200	1.08031700	-0.48621100
263	O	6.17601000	2.42382400	-0.59043000
264	O	6.50244600	0.35108500	0.45703400
265	H	7.41848700	0.34184700	0.16251700
266	C	-3.43955300	2.54950200	0.40626900
267	C	-3.40866400	1.36693200	-0.55856200
268	H	-3.19528200	1.71715200	-1.58844900
269	H	-4.40666700	0.91559300	-0.58157800
270	H	6.86172600	2.47059100	-1.26249000
271	H	5.81683700	0.59680500	-1.47284200
272	H	-0.97847100	1.78944200	-0.45054100

273	H	-0.91849600	0.46874500	-1.62411100
274	C	-2.81519200	-0.96418900	-0.73024000
275	C	-3.90882300	-1.66736300	0.06801800
276	C	-4.29621500	-3.00765300	-0.54710800
277	N	-5.35936400	-3.63598000	0.24550200
278	H	-3.12753000	-0.84652100	-1.78712500
279	H	-1.92282900	-1.59990400	-0.73741800
280	H	-4.80709700	-1.04511200	0.13728300
281	H	-3.54142800	-1.81925500	1.09080000
282	H	-3.39270900	-3.63184400	-0.64242900
283	H	-4.68586000	-2.84446100	-1.55827300
284	H	-5.01566600	-3.83432900	1.18093000
285	H	-5.62712000	-4.52670100	-0.16264800
286	N	-4.54258900	4.79071800	0.85398100
287	C	-4.42788700	3.63268900	-0.03641500
288	H	-4.82421900	4.48569700	1.78135200
289	H	-3.63567300	5.23654700	0.95940000
290	H	-4.14471300	4.00272400	-1.02866000
291	H	-5.42675000	3.19264900	-0.13934700
292	H	-2.44109400	2.99559000	0.49739300
293	H	-3.71622900	2.17740900	1.40033000
294	C	1.93407000	-3.18564500	0.31570100

295	O	1.48154500	-2.96341400	-0.74265000
296	O	2.37903500	-3.47555000	1.35790100
297	AA-Pro			
298	C	-0.49622800	0.00505000	-0.33475000
299	C	0.58978400	0.99959600	-0.73942700
300	C	0.62351600	2.25235100	0.17660800
301	C	2.11614100	2.50664200	0.41652100
302	C	2.65632200	1.07439000	0.43994300
303	N	1.93686600	0.37108900	-0.63831300
304	H	2.43096500	0.53527300	-1.51131600
305	H	0.41768800	1.30035300	-1.78236200
306	H	0.10175000	3.10594500	-0.26553800
307	H	0.13585900	2.01866700	1.13186900
308	H	2.31240300	3.06711100	1.33575900
309	H	2.55449300	3.05421900	-0.42657100
310	H	2.41435100	0.59516600	1.39644600
311	H	3.73651000	0.99591900	0.28687700
312	H	-0.31487500	-0.31634700	0.69800900
313	C	-2.98678200	-0.41655000	-0.07951400
314	C	-1.90243000	0.58835200	-0.45393400
315	O	-4.28315400	0.14802800	-0.23410100
316	O	-2.88226400	-0.84188300	1.24728300

317	H	-2.89066700	-1.32697000	-0.68335500
318	H	-2.02349900	1.46645700	0.19412900
319	H	-2.07121300	0.92212200	-1.48867300
320	H	-0.41386700	-0.88352200	-0.97269100
321	H	-4.29516900	0.62489100	-1.06976900
322	H	-3.11185600	-0.07967500	1.79189400
323	C	1.87229600	-2.44108300	0.04698500
324	O	2.05325700	-2.15025500	1.16649300
325	O	1.69007900	-2.79210400	-1.05481200
326	AA-His			
327	C	-1.68850400	-0.61405600	0.37579100
328	N	-2.15214700	0.66665800	0.15538300
329	C	-2.76326900	-1.42849100	0.05027900
330	C	-3.44660500	0.57615600	-0.27626300
331	N	-3.85002100	-0.68605300	-0.35157800
332	H	-2.80266300	-2.50871300	0.09838500
333	H	-4.04274800	1.44761100	-0.50926900
334	H	-1.61050400	1.51291400	0.28231400
335	C	-0.29192400	-0.91207500	0.81212000
336	C	0.69026700	-0.98786400	-0.36530800
337	C	2.11974700	-1.09810900	0.13966400
338	O	2.98855200	-1.17257200	-0.97808000

H	0.05172000	-0.14002300	1.51466600
H	-0.27636800	-1.86618600	1.34542200
N	0.59130400	0.22942300	-1.17736500
H	0.48621400	-1.90317800	-0.94202700
O	2.18641500	-2.26374400	0.92185800
H	2.37332300	-0.20878500	0.73422300
H	3.74916500	-0.61139900	-0.80359200
H	3.11116800	-2.41256800	1.14342700
H	-0.32240900	0.24898500	-1.62559100
H	1.27588900	0.16337000	-1.92849700
C	1.21257500	2.66705100	0.27412600
O	2.35711700	2.50945400	0.09835700
O	0.07910900	2.87587500	0.48824300

PA-EDA

C	-1.65511700	-1.15216100	0.00148100
C	-3.09222600	-0.66564600	0.13295300
H	-1.38667000	-1.16180300	-1.05907400
H	-1.58728000	-2.19509100	0.36562400
H	-3.16420900	0.34007300	-0.28977700
H	-3.34420800	-0.58913900	1.20411300
N	-0.74428000	-0.26353800	0.72331800
H	-1.08871200	-0.17677000	1.67834200

361	N	-3.98393200	-1.55252300	-0.61846800
362	H	-4.92825100	-1.18080000	-0.62867700
363	H	-4.03079700	-2.46330700	-0.17140500
364	C	1.26899900	-0.78192700	-0.60687400
365	C	0.62923000	-0.78144500	0.78368500
366	H	0.85721400	0.05055700	-1.18838700
367	H	1.04664400	-1.70529800	-1.15143900
368	H	1.20476300	-0.12368700	1.44111900
369	H	0.67241800	-1.79646200	1.20831600
370	C	2.77763100	-0.61618800	-0.55121100
371	O	3.29973700	-1.65678300	0.22827500
372	O	3.16947700	0.60161700	0.06642600
373	H	2.64623600	1.31230300	-0.32070900
374	H	4.23278700	-1.45828500	0.36256000
375	H	3.19959200	-0.65053700	-1.56746900
376	C	-0.57345000	2.56517700	-0.06329500
377	O	-1.70622100	2.72636800	0.17370500
378	O	0.56670000	2.46677500	-0.31809600
379	7-Azaindole			
380	H	-1.98760800	-2.71918500	-0.15589700
381	N	-1.92461300	1.73262400	-0.01202100
382	C	-0.89434100	2.63493100	-0.13186500

383	C	0.30298000	1.96042600	-0.31067800
384	C	-0.01051300	0.56387700	-0.29664300
385	C	-1.41989600	0.45666200	-0.11791800
386	C	0.71764000	-0.63509000	-0.45524100
387	C	-0.02801700	-1.81325200	-0.39311700
388	C	-1.42672100	-1.78743000	-0.19856000
389	N	-2.14792500	-0.66521200	-0.05425000
390	H	-2.90617400	1.94105800	0.11286700
391	H	-1.08738400	3.69868000	-0.08611700
392	H	1.27241500	2.42128100	-0.44422100
393	H	0.46885700	-2.77449100	-0.50556000
394	C	2.21392800	-0.62043600	-0.59029900
395	C	2.86961900	-0.36671600	0.77517000
396	C	4.36479500	-0.12147000	0.66842200
397	H	2.56827800	-1.57363500	-0.99276700
398	H	2.52020500	0.16861500	-1.28655400
399	H	2.69871600	-1.22481000	1.43616600
400	H	2.41164700	0.50796000	1.25335100
401	O	4.93582600	-1.21289400	0.00847900
402	H	4.80253100	0.00006100	1.67237500
403	O	4.66272700	1.04031200	-0.10321000
404	H	5.84817300	-0.97122500	-0.18447300

405	H	4.58437800	1.80171600	0.48023700
406	C	-4.95930700	-0.41397100	0.28023400
407	O	-4.85276500	0.75451500	0.30461100
408	O	-5.12919900	-1.56856900	0.26355300
409	TBD			
410	H	-1.16284500	-1.75515500	-1.84941400
411	N	-2.25025800	1.45916500	0.52348000
412	H	-3.21807800	1.15710700	0.47225800
413	C	-2.04683500	2.83674800	0.08247200
414	H	-2.63480800	3.49285100	0.73088700
415	C	-0.56497700	3.15712600	0.17234000
416	H	-0.24202700	3.12362000	1.21784700
417	C	0.20749500	2.11853600	-0.62723900
418	H	1.28004100	2.29360400	-0.53009200
419	N	-0.09125200	0.76784500	-0.13872600
420	C	-1.45061600	0.44761900	-0.02204800
421	N	-2.01639000	-0.69261900	-0.25321600
422	C	-1.16569000	-1.76584700	-0.74908700
423	H	0.88608400	-2.43706900	-0.64860400
424	C	0.25770400	-1.64423300	-0.23067500
425	C	0.83440800	-0.28076500	-0.59434000
426	H	-2.38966600	2.98913900	-0.95468900

427	H	-0.36514300	4.15656200	-0.22865900
428	H	-0.04484800	2.20364900	-1.69958800
429	H	-1.60927700	-2.71864000	-0.44181200
430	H	0.25581000	-1.74544800	0.86306400
431	H	0.94783900	-0.22250700	-1.69358500
432	C	2.20918200	-0.07508600	0.05771400
433	C	3.24432400	-1.10236700	-0.40181800
434	C	4.66668000	-0.79712000	0.07146800
435	H	2.58846600	0.92040100	-0.19104100
436	H	2.07388600	-0.11027300	1.14517100
437	H	3.26842100	-1.14491900	-1.49817000
438	H	2.98296200	-2.10724200	-0.04676100
439	O	5.13878900	0.44370800	-0.37156600
440	H	5.35845500	-1.54030800	-0.34309200
441	O	4.75251100	-0.77966200	1.48978700
442	H	4.28126000	-1.54848600	1.82589800
443	H	4.79309300	1.09811400	0.24567300
444	C	-4.64609800	-1.42570600	0.32183200
445	O	-5.00170500	-0.31795400	0.46661000
446	O	-4.36681900	-2.55348700	0.19269800
447	Aminoterephthalicacid			
448	C	-0.81777200	0.82407500	1.07432600

449	C	-2.19790600	0.76217300	0.82802600
450	C	-2.67899700	1.12164100	-0.44191000
451	C	-1.79018100	1.55036800	-1.43201600
452	C	-0.41462800	1.60289200	-1.17742600
453	C	0.08056400	1.24285700	0.08496900
454	H	-0.43751400	0.52828800	2.05261200
455	N	-3.06792800	0.22202000	1.79491600
456	H	-2.17511400	1.83709700	-2.40760600
457	H	0.27357200	1.93256300	-1.95290900
458	H	-3.74594200	1.06736900	-0.64852800
459	H	-4.02675600	0.53204800	1.69490800
460	H	-2.75209200	0.36250600	2.74671000
461	C	1.56461000	1.20938100	0.35129400
462	C	2.12134400	-0.20651100	0.15423800
463	C	3.62205000	-0.28015800	0.36439200
464	H	2.08067000	1.90405200	-0.31975600
465	H	1.76695600	1.53954700	1.37860700
466	H	1.89513400	-0.55927900	-0.86106100
467	H	1.63412400	-0.90124500	0.84667000
468	O	3.98591100	-1.64149100	0.27613100
469	H	3.90323900	0.13797000	1.33853600
470	O	4.34110000	0.49967700	-0.56868200

471	H	4.94285100	-1.67535400	0.38436000
472	H	4.08617900	0.17649500	-1.44136100
473	C	-2.25147800	-2.09685600	-0.53547200
474	O	-1.12588500	-2.16835300	-0.22324800
475	O	-3.37555800	-2.04175400	-0.85896600
476	Diamine			
477	C	-2.39529400	-0.12209000	0.01834000
478	C	-1.47617200	0.25076500	-1.14755100
479	N	-0.31354700	1.04989100	-0.71033200
480	C	-0.64433800	1.92392800	0.42933500
481	C	-2.10210600	2.36806300	0.35931600
482	C	-3.03917600	1.16324300	0.57726900
483	H	-3.99864900	1.34698600	0.08099800
484	H	-3.24936100	1.04251400	1.64599500
485	H	-2.27837100	2.82409000	-0.62123000
486	H	-2.28723400	3.14336200	1.10971400
487	H	-1.80188400	-0.61420100	0.79937500
488	H	-3.15893300	-0.83881100	-0.30414000
489	H	-1.09944000	-0.63636700	-1.66474700
490	H	-2.04766800	0.82742300	-1.88584700
491	H	0.02377100	2.79090200	0.42059600
492	H	-0.47853800	1.39461800	1.38959300

493	C	0.82960900	0.20215300	-0.39208100
494	C	2.10669200	1.01531800	-0.20209800
495	N	3.22267900	0.07999300	-0.05192500
496	H	0.64786600	-0.39969200	0.52405100
497	H	0.99190800	-0.48905600	-1.22598800
498	H	2.03450100	1.68196300	0.66692200
499	H	2.28140500	1.62252900	-1.09423300
500	O	4.38214700	0.85250400	0.31433000
501	H	3.04076500	-0.47676700	0.78290100
502	H	5.01811600	0.58671700	-0.35653600
503	C	-0.39700600	-3.15253700	0.21351700
504	O	-0.69978400	-3.27322100	-0.91046400
505	O	-0.09429300	-3.03034000	1.33764600
506	1,2-Aminoterephthalicacid			
507	C	-0.38177300	-0.32238700	-0.54536600
508	C	-1.61006000	0.25267400	-0.88998100
509	C	-2.06030800	1.39950700	-0.19979800
510	C	-1.21397700	2.00132800	0.73787800
511	C	0.02721400	1.43439400	1.05274300
512	C	0.45855800	0.26350500	0.41392900
513	H	-0.06346900	-1.23038600	-1.05887700
514	N	-2.49293900	-0.35555300	-1.80865700

515	H	-1.55390800	2.89345900	1.26155700
516	H	0.66174600	1.90713300	1.79970800
517	N	-3.30866800	1.94602700	-0.57603600
518	H	-2.98532800	0.33737400	-2.36631900
519	H	-2.02441700	-1.01708800	-2.41768900
520	C	1.81132900	-0.33519000	0.70842500
521	C	2.82579100	0.00963300	-0.38871600
522	C	4.21395000	-0.52997300	-0.09880300
523	H	2.17666200	0.03279800	1.67304100
524	H	1.72532200	-1.42682400	0.79184400
525	H	2.89527800	1.09995200	-0.50287900
526	H	2.49042300	-0.39282100	-1.35062600
527	O	5.01048300	-0.25100300	-1.23153100
528	H	4.18225300	-1.60767000	0.10148100
529	O	4.78037100	0.02432200	1.06999900
530	H	5.89542100	-0.57406800	-1.02881600
531	H	4.78367500	0.98036800	0.94015700
532	H	-3.56933200	2.73110400	0.01035600
533	H	-4.04545400	1.24635200	-0.52846900
534	C	-3.30070900	-1.78303800	0.71540300
535	O	-4.00543100	-0.89375900	1.00507100
536	O	-2.60763800	-2.68652700	0.44822100

Single-molecule calculations with H_2O

AA-Gly

C	1.34058200	-0.07210200	0.30317200
O	1.71750100	1.09627900	-0.39202800
O	2.05741300	-1.19946200	-0.15406700
H	2.72879500	-1.41860800	0.49660500
C	-0.13200200	-0.29848700	0.00531600
H	-0.47850300	-1.16562400	0.57312600
H	-0.21374500	-0.54530600	-1.05719200
N	-0.99891500	0.84432000	0.29599900
H	-0.64891700	1.65851000	-0.20221300
H	-0.95290600	1.07398700	1.28598300
H	2.67741000	1.15526300	-0.36685600
H	1.50837300	0.06291300	1.38302400
O	-3.52454900	-0.47742100	-0.12194400
H	-2.74463800	0.10087100	-0.04079600
H	-4.13785400	0.03612700	-0.65029000

AA-Arg

C	-3.73937500	0.77025200	0.09474900
O	-2.86081800	1.85312500	-0.01952000
O	-3.99687100	0.54162500	1.46013200
H	-4.32424200	-0.37056400	1.49702800

559	C	-3.10447700	-0.46244500	-0.55817700
560	H	-3.23711000	2.56433100	0.51049900
561	H	-4.68954900	0.99670700	-0.41953700
562	C	-1.73707000	-0.80497400	0.04206200
563	C	-0.57241100	-0.08985800	-0.64425800
564	C	0.75728900	-0.42882200	0.01983000
565	N	1.84452300	0.23957200	-0.68491200
566	H	-1.74914900	-0.57177900	1.11240900
567	H	-0.73807600	0.99081400	-0.60425100
568	H	0.92745800	-1.50980400	-0.02886900
569	C	3.17893500	0.08202000	-0.34137800
570	N	-4.05032900	-1.55943600	-0.29207600
571	H	-3.63220800	-2.45339200	-0.53023600
572	H	-4.88477600	-1.46199500	-0.86633600
573	H	-2.98823800	-0.25131500	-1.63205100
574	H	-1.58191500	-1.88916900	-0.04790400
575	H	-0.52206100	-0.38437900	-1.69984700
576	H	0.71288800	-0.13568600	1.08138200
577	H	1.62056000	1.17574800	-0.99585300
578	N	3.45619200	-1.00564300	0.46746400
579	N	4.15558600	0.79987000	-0.80674700
580	H	3.80708100	1.50191200	-1.45644700

581	H	4.41690200	-0.99543800	0.80180500
582	H	2.78639100	-1.18943100	1.20034800
583	O	6.20833700	0.08101900	0.91990100
584	H	7.05715400	-0.07098800	0.49982000
585	H	5.67455700	0.53071000	0.23085900
586	Melamine			
587	C	2.54085300	1.30144600	0.12377600
588	N	1.25451000	1.65342800	0.08433600
589	C	0.40929300	0.60242000	0.01591000
590	N	0.74993400	-0.69419900	0.02500100
591	C	2.07569100	-0.90666700	0.08648600
592	N	3.02772400	0.04779000	0.11998300
593	N	3.46432700	2.31590200	0.12198900
594	N	-0.91023300	0.91144800	-0.10032300
595	N	2.49141800	-2.19674800	0.16792700
596	H	3.09915700	3.21893500	0.38737900
597	H	4.37002300	2.07660000	0.49792300
598	H	-1.11695300	1.88137000	0.10164900
599	H	1.80448300	-2.89606100	-0.06701300
600	H	3.46762700	-2.38621300	-0.03072900
601	C	-1.96433600	-0.06766100	0.12412300
602	C	-3.28973600	0.48989000	-0.38227100

603	C	-4.42769800	-0.49965600	-0.21806800
604	H	-1.69613100	-0.97604600	-0.42113200
605	H	-2.04530000	-0.32906800	1.18558500
606	H	-3.19142300	0.74427500	-1.44436100
607	H	-3.56870900	1.39839300	0.16419500
608	O	-4.56927000	-0.76564700	1.14552800
609	H	-4.21208500	-1.42768300	-0.77336900
610	O	-5.66619300	0.03254500	-0.66893800
611	H	-5.38120400	-1.27470600	1.24561800
612	H	-5.61271100	0.12729600	-1.62554800
613	O	5.34877600	-1.50351900	-0.37873700
614	H	4.74722300	-0.74773800	-0.24683800
615	H	5.82133000	-1.29836900	-1.18828500
616	AA-Lys			
617	C	2.76484900	-0.63675800	-0.49651000
618	O	1.85562100	-1.24582500	-1.36713200
619	O	2.93988000	-1.46612100	0.62815100
620	H	3.27907500	-0.87296200	1.31632100
621	C	2.22468800	0.73759600	-0.08696700
622	H	2.13413900	-2.16443900	-1.44959600
623	H	3.73929300	-0.50247500	-0.99886500
624	C	0.83054500	0.66234800	0.54414300

C	-0.31419100	0.75130500	-0.46724600
C	-1.68102500	0.68601900	0.21369100
C	-2.82827700	0.79921600	-0.78353000
H	0.74536300	-0.26273600	1.12451500
H	-0.22201600	-0.06603100	-1.18776000
H	-1.76287800	1.50113200	0.94833700
N	-4.12617100	0.70849300	-0.08627600
H	-2.77488200	-0.03418300	-1.49189000
N	3.19249100	1.24260500	0.90360800
H	2.82367800	2.06613800	1.36867200
H	4.05969400	1.52173300	0.45016600
H	2.18014700	1.36229500	-0.99220600
H	0.73020900	1.49490000	1.25548500
H	-0.22710900	1.69344600	-1.02738100
H	-1.78057800	-0.25781800	0.76098100
H	-4.88791400	0.84715800	-0.74603400
H	-4.19467600	1.46370900	0.59203300
H	-2.72319100	1.73239900	-1.35645900
O	-3.79107200	-2.00480200	0.78201900
H	-4.55022100	-2.35854500	1.24891300
H	-4.04733600	-1.08576800	0.57765700

PA-DAP

647	C	-1.24220100	-0.48767000	0.12626600
648	C	-2.70699000	-0.11049900	0.31304300
649	H	-0.93115700	-0.23038200	-0.89203100
650	H	-1.11398000	-1.57767200	0.24573900
651	H	-2.81011200	0.97589500	0.20229700
652	H	-3.02619800	-0.36880100	1.33308200
653	N	-0.39140300	0.25516700	1.06575600
654	H	-0.78895000	0.14130300	1.99713300
655	C	-3.62589800	-0.81497800	-0.69030100
656	N	-5.05052500	-0.50042400	-0.57524000
657	H	-3.30931200	-0.55972000	-1.70775500
658	C	1.70697200	0.00712500	-0.22774000
659	C	0.99569300	-0.24093300	1.10124000
660	H	1.48306700	1.02371500	-0.57043300
661	H	1.38042200	-0.70111700	-0.99543200
662	H	1.51614700	0.30516200	1.89744500
663	H	1.04053200	-1.31225400	1.34571200
664	C	3.20900200	-0.13904700	-0.09498700
665	O	3.87535100	0.02683000	-1.33805600
666	O	3.48217800	-1.43877100	0.34545900
667	H	4.43641300	-1.55581700	0.28157300
668	H	-5.19782600	0.49753500	-0.69412800

H	-5.38824400	-0.73866100	0.35259700
H	-3.51408100	-1.90037200	-0.58768600
H	3.68253000	0.91651900	-1.65254800
H	3.60194000	0.59913300	0.62409700
O	-0.13748700	2.83136800	-0.09261000
H	-0.44181800	3.54387200	0.47335400
H	-0.30567900	2.01905600	0.42990000
PA-TAEA			
C	-0.15465700	-0.22395100	0.24353300
C	1.28261500	-0.42555000	-0.23266700
H	-0.37261600	0.84994400	0.32382600
H	-0.25168900	-0.65529200	1.24708200
H	1.37459700	-0.07607800	-1.27654900
H	1.49621500	-1.49727300	-0.23988300
N	-1.07361100	-0.94442200	-0.64328500
H	-1.05037500	-0.51823300	-1.56851600
N	2.23910100	0.27033800	0.63187500
C	3.22902700	-0.60259700	1.26746200
C	-3.10788600	0.39517500	0.04940900
C	-2.46248100	-0.97474700	-0.16866400
H	-3.03883200	0.99760900	-0.86493800
H	-2.61153100	0.94118700	0.85660400

691	H	-3.04955800	-1.53955100	-0.89905900
692	H	-2.46915200	-1.54619200	0.76867400
693	C	-4.57601300	0.28125900	0.39885900
694	O	-5.01854200	1.57235400	0.75553700
695	O	-5.26945200	-0.20926400	-0.73549900
696	H	-5.97540000	-0.78699700	-0.43368000
697	N	4.00101600	-1.78422700	-0.77602700
698	C	4.42496800	-1.02847000	0.41031600
699	H	4.81577000	-2.17799500	-1.23896900
700	H	3.57159700	-1.15747600	-1.45124100
701	H	5.03315100	-0.14395600	0.16669000
702	H	5.04984300	-1.68752800	1.02407700
703	C	1.79998100	2.57117800	-0.18377100
704	C	2.82350200	1.45893100	0.02772700
705	N	2.47689400	3.74369300	-0.74744100
706	H	1.28861000	2.75657800	0.77204200
707	H	1.04448700	2.24574000	-0.90668200
708	H	3.60101600	1.83144500	0.70856100
709	H	3.31185100	1.25679600	-0.94415600
710	H	3.08534300	4.16068300	-0.04874600
711	H	1.79989000	4.45456700	-1.00585200
712	H	2.70273200	-1.49601700	1.61969700

713	H	3.61866100	-0.07843100	2.15098800
714	H	-5.97747600	1.53897600	0.83143300
715	H	-4.72380100	-0.41379800	1.23954500
716	O	-0.36611500	-3.64955600	-0.03221900
717	H	0.45596400	-3.90542600	-0.45646900
718	H	-0.55456500	-2.76659800	-0.40609800
719	PA-TAPA			
720	C	-1.55375000	-0.10204200	0.20267200
721	C	-0.13103900	-0.24862200	-0.32671000
722	H	-1.81270500	0.96635500	0.27168800
723	H	-1.61627100	-0.52275900	1.21485800
724	H	-0.06212200	0.16555200	-1.34072000
725	H	0.10898400	-1.31333800	-0.39016200
726	N	-2.49124300	-0.86117600	-0.63489300
727	H	-2.45128900	-0.49716000	-1.58577400
728	C	0.88163200	0.47298400	0.55806000
729	N	2.25917900	0.20851500	0.13485900
730	C	-4.52327700	0.55316700	-0.08356300
731	C	-3.88276500	-0.83511300	-0.16681200
732	H	-4.49856300	1.04337400	-1.06470800
733	H	-3.99030900	1.19086800	0.62672600
734	H	-4.47256600	-1.46349900	-0.83962600

735	H	-3.89874300	-1.31193500	0.82237200
736	C	-5.97057000	0.49592300	0.35624700
737	O	-6.47141300	1.81838300	0.37889200
738	O	-6.65671100	-0.31270100	-0.57641600
739	H	-7.58912200	-0.31479700	-0.33835600
740	C	3.11130100	2.50674500	-0.30735500
741	C	3.15666100	1.27366100	0.59152400
742	H	2.92885500	1.55435000	1.63961700
743	H	4.17999100	0.88255000	0.58748700
744	H	-7.15215300	1.87316100	1.05521500
745	H	-6.05412900	0.05637200	1.36207700
746	H	0.70136700	1.55227300	0.50118400
747	H	0.73660500	0.18063900	1.61747500
748	C	2.70605400	-1.09564900	0.63894100
749	C	3.83358500	-1.68739400	-0.20119500
750	C	4.30855700	-3.02962100	0.34412000
751	N	5.39434900	-3.55612400	-0.49228900
752	H	3.02006200	-1.01354200	1.69863500
753	H	1.85489000	-1.78449500	0.61616100
754	H	4.69089400	-1.00751800	-0.24671900
755	H	3.46478100	-1.81273800	-1.22699400
756	H	3.44502800	-3.70943400	0.42068400

757	H	4.70477400	-2.89096100	1.35644400
758	H	5.04457800	-3.73245600	-1.42998200
759	H	5.71827500	-4.44820400	-0.13017300
760	N	4.07565700	4.83104800	-0.63607700
761	C	4.03546200	3.62183700	0.19091000
762	H	4.37062400	4.59329800	-1.57889000
763	H	3.14257000	5.22516900	-0.71633500
764	H	3.73630900	3.92034800	1.20237200
765	H	5.05943000	3.23775700	0.26748800
766	H	2.08746800	2.89655400	-0.37166600
767	H	3.40417200	2.20632000	-1.32084400
768	O	-1.33394300	-3.37075500	0.02613800
769	H	-1.80204000	-4.12035700	-0.34736100
770	H	-1.79980600	-2.59226000	-0.34332200
771	AA-Pro			
772	C	0.28433000	0.31703200	-0.25662300
773	C	-0.95365400	-0.43397000	-0.74310800
774	C	-1.15548200	-1.78890900	-0.00572700
775	C	-2.63107000	-1.78471900	0.41824700
776	C	-2.87991300	-0.29044800	0.63508400
777	N	-2.18013500	0.37437400	-0.48198600
778	H	-2.78056100	0.30616500	-1.30151200

779	H	-0.86879300	-0.59082000	-1.82606100
780	H	-0.89084700	-2.64644100	-0.63072300
781	H	-0.51898800	-1.81834600	0.88717200
782	H	-2.82096600	-2.39617600	1.30546800
783	H	-3.26994600	-2.14713900	-0.39579100
784	H	-2.42581700	0.03623300	1.57858400
785	H	-3.93358800	0.00003600	0.64560600
786	H	0.20018600	0.47716700	0.82577600
787	C	2.80887200	0.29697500	-0.02054600
788	C	1.58112500	-0.42991800	-0.56020200
789	O	4.00407700	-0.39220300	-0.36255100
790	O	2.78810500	0.40581800	1.37337000
791	H	2.83864800	1.32809300	-0.39250800
792	H	1.57375000	-1.43754600	-0.12414700
793	H	1.68576200	-0.54690900	-1.64888400
794	H	0.30859000	1.30504400	-0.73072900
795	H	3.94129500	-0.64462800	-1.28894800
796	H	2.95048000	-0.48281200	1.71155300
797	O	-1.77770500	3.21308300	-0.07986200
798	H	-1.91319300	2.25539900	-0.21574900
799	H	-1.25612400	3.25221900	0.72438800

800 AA-His

801	C	0.67880000	-0.27484100	-0.59065200
802	N	1.21046500	0.93542900	-0.98583700
803	C	1.76279600	-0.97927600	-0.09188000
804	C	2.54719400	0.92540600	-0.71404000
805	N	2.91498900	-0.23041500	-0.17215900
806	H	1.76625200	-1.98302900	0.31076400
807	H	3.19847000	1.76259100	-0.92251600
808	H	0.66592800	1.70801500	-1.34818300
809	C	-0.76882500	-0.60534400	-0.73350100
810	C	-1.64430500	0.06884200	0.33261100
811	C	-3.11324800	-0.10149600	-0.01853900
812	O	-3.87204500	0.50403300	1.01158400
813	H	-1.12128300	-0.28871900	-1.72454400
814	H	-0.89555900	-1.68884700	-0.67006700
815	N	-1.35191100	1.50556300	0.38267100
816	H	-1.48969000	-0.43404400	1.29961700
817	O	-3.35199900	-1.48359900	-0.11950200
818	H	-3.32206600	0.40360400	-0.97345900
819	H	-4.70947000	0.79723000	0.64114300
820	H	-4.26239200	-1.61215000	-0.40309400
821	H	-0.46647000	1.64407400	0.86375200
822	H	-2.05836100	1.95006500	0.96500200

O	5.46316200	-0.34758100	1.21238100
H	4.60310600	-0.35746800	0.75652700
H	5.98931800	-0.95792600	0.69264400

7-Azaindole

H	-2.20975000	-3.13656100	0.04039800
N	-2.66173600	1.29924200	0.03878400
C	-1.74515500	2.30484200	-0.14268000
C	-0.47995100	1.76767800	-0.32879600
C	-0.62945100	0.34700400	-0.25875100
C	-2.01395300	0.08989000	-0.03627400
C	0.22754600	-0.76641400	-0.39268300
C	-0.37567400	-2.01962900	-0.27346100
C	-1.76239700	-2.14850000	-0.04449800
N	-2.60325300	-1.10999500	0.08302300
H	-3.66632700	1.38058100	0.17982500
H	-2.05851100	3.34054900	-0.13065200
H	0.42605100	2.33136900	-0.50568900
H	0.22582400	-2.92110400	-0.36718000
C	1.70848000	-0.58678200	-0.57010000
C	2.36181600	-0.19891200	0.76460900
C	3.81788800	0.20377600	0.60673600
H	2.16122100	-1.51012000	-0.94237200

H	1.90475700	0.20049400	-1.30684100
H	2.30237300	-1.03879100	1.46714700
H	1.81999400	0.64134500	1.21599700
O	4.49299400	-0.85011600	-0.01507600
H	4.26015200	0.42294100	1.59196700
O	3.96896900	1.35045900	-0.22716700
H	5.36717400	-0.51809900	-0.24661900
H	3.81469900	2.12731000	0.31966000
O	-5.18869500	0.04029400	0.32147100
H	-4.50927400	-0.65867400	0.28293700
H	-5.82449800	-0.26878700	0.97033100

TBD

H	-1.29861400	-2.37753300	-1.55188600
N	-2.85373800	0.79457300	0.59787000
H	-3.76960200	0.35041300	0.61767300
C	-2.88274900	2.16677000	0.10145400
H	-3.53966000	2.74893100	0.75401700
C	-1.46945600	2.72092800	0.10630000
H	-1.10120500	2.79437500	1.13471900
C	-0.57711500	1.77382400	-0.68077800
H	0.45623800	2.12156900	-0.65220900
N	-0.63065200	0.42528600	-0.10536800

867	C	-1.90733600	-0.10337000	0.11045000
868	N	-2.26186300	-1.34884300	0.00490000
869	C	-1.25755600	-2.29638700	-0.45536400
870	H	0.88182000	-2.59128300	-0.41380600
871	C	0.14008800	-1.88773200	-0.02289400
872	C	0.45182000	-0.48075700	-0.51886800
873	H	-3.29144200	2.21834300	-0.92089600
874	H	-1.44796000	3.71644000	-0.34971300
875	H	-0.88939400	1.75197700	-1.73977800
876	H	-1.50833500	-3.28367900	-0.05323000
877	H	0.19882800	-1.89634800	1.07384100
878	H	0.51863300	-0.49770400	-1.62302100
879	C	1.78973100	0.01350900	0.05047700
880	C	2.97015200	-0.86844200	-0.35820300
881	C	4.33383100	-0.27650700	0.00280700
882	H	1.98941600	1.02804600	-0.30587000
883	H	1.69332300	0.06342200	1.14153200
884	H	2.96171400	-1.02608200	-1.44426400
885	H	2.90096800	-1.85793800	0.11109600
886	O	4.56734400	0.96764700	-0.59422700
887	H	5.12864000	-0.93551200	-0.36713500
888	O	4.46474500	-0.08300900	1.40432300

889	H	4.15504900	-0.88023100	1.84549800
890	H	4.14970000	1.62196300	-0.02312800
891	O	-5.03450600	-1.29126400	0.22712500
892	H	-4.09161400	-1.55220300	0.13513700
893	H	-5.40185800	-1.93208800	0.83956000
894	Aminoterephthalicacid			
895	C	-1.21440900	0.02769400	0.82411900
896	C	-2.55513600	0.11230700	0.42325400
897	C	-2.93869700	1.11396100	-0.48110700
898	C	-1.99687500	2.04283900	-0.93300300
899	C	-0.66393300	1.96322100	-0.51369300
900	C	-0.26336700	0.95445100	0.37525200
901	H	-0.91133800	-0.76626800	1.50701600
902	N	-3.47654700	-0.88555100	0.82854400
903	H	-2.30515800	2.82568500	-1.62121300
904	H	0.06422700	2.69065300	-0.86579900
905	H	-3.97432700	1.17941200	-0.80865100
906	H	-4.44039300	-0.57505000	0.77400800
907	H	-3.29106800	-1.23015700	1.76454500
908	C	1.18254300	0.80451900	0.77716000
909	C	1.87701700	-0.26847700	-0.07069500
910	C	3.34835900	-0.42465100	0.26748600

H	1.70340500	1.76024700	0.65902200
H	1.24783800	0.52799900	1.83747000
H	1.79259900	-0.01403200	-1.13598500
H	1.38277200	-1.23595100	0.07023400
O	3.83404700	-1.50115900	-0.50457400
H	3.48580900	-0.61514300	1.33863700
O	4.08997900	0.75255500	0.02533500
H	4.76945600	-1.59240400	-0.29189100
H	3.97312200	0.95619400	-0.91054900
O	-2.33178900	-3.08718600	-0.82332200
H	-1.72036200	-2.57625300	-1.35860300
H	-2.83166300	-2.40493600	-0.35019500
Diamine			
C	2.36564000	-1.37418500	0.63812500
C	1.48500400	-0.29449000	1.26630800
N	0.39852200	0.14707100	0.35756800
C	0.79356000	0.04590000	-1.06474600
C	2.28102300	0.34384200	-1.21748400
C	3.11714400	-0.77662700	-0.56826300
H	4.08394700	-0.37557600	-0.24475600
H	3.32616000	-1.56299200	-1.30225300
H	2.48635100	1.31186300	-0.74803200

933	H	2.52655500	0.44656200	-2.27910800
934	H	1.73182300	-2.21085500	0.31990500
935	H	3.06478200	-1.77275300	1.38118000
936	H	1.02159800	-0.64394800	2.19430600
937	H	2.10248900	0.57637800	1.52063900
938	H	0.19577000	0.75877100	-1.64101000
939	H	0.57671900	-0.96605200	-1.45543400
940	C	-0.84100700	-0.58881000	0.60805100
941	C	-2.00546200	-0.03594500	-0.21007500
942	N	-3.24118400	-0.62373100	0.30994100
943	H	-0.71501800	-1.66815600	0.38886500
944	H	-1.09095700	-0.48856300	1.66971000
945	H	-1.88632200	-0.25505100	-1.27869400
946	H	-2.05971800	1.04907300	-0.08322500
947	O	-4.29502800	-0.28099100	-0.60922000
948	H	-3.16346500	-1.63556700	0.20826000
949	H	-4.93988400	0.13390700	-0.02839000
950	O	0.43328200	2.95722400	0.39717900
951	H	0.03814000	3.32384700	1.19101100
952	H	0.31822100	1.98776100	0.49929900
953	1,2-Aminoterephthalicacid			
954	C	-0.57966800	-0.72296800	0.20243300

955	C	-1.87380600	-0.52996600	-0.28928700
956	C	-2.37513200	0.77838500	-0.46380800
957	C	-1.51171100	1.85808400	-0.23719600
958	C	-0.20772900	1.65538600	0.22927200
959	C	0.27617900	0.35969800	0.45644700
960	H	-0.22004700	-1.74121700	0.35711800
961	N	-2.75147200	-1.62194000	-0.53426900
962	H	-1.88566000	2.87019400	-0.38294100
963	H	0.43650200	2.51214600	0.41690200
964	N	-3.68312700	0.93121000	-0.97618600
965	H	-3.30982700	-1.43698600	-1.36737000
966	H	-2.23694100	-2.48651300	-0.67022100
967	C	1.69508000	0.12233900	0.90958200
968	C	2.59183700	-0.30254000	-0.25974800
969	C	4.04160200	-0.49091400	0.14633400
970	H	2.09526800	1.03461500	1.36446500
971	H	1.71459500	-0.65746000	1.68254300
972	H	2.55065500	0.45418000	-1.05491300
973	H	2.22736700	-1.24099500	-0.69132600
974	O	4.72429100	-0.99778400	-0.98180700
975	H	4.12515000	-1.17937500	0.99584500
976	O	4.63753100	0.70336800	0.60694900

977	H		5.64855600	-1.08602600	-0.72385400
978	H		4.53949800	1.34589000	-0.10610200
979	H		-3.92805900	1.90861200	-1.08721100
980	H		-4.37925000	0.48323100	-0.38034100
981	O		-4.96354400	-0.87819000	1.11039100
982	H		-5.62677100	-1.50553900	1.40548100
983	H		-4.23497000	-1.42382400	0.76669300

984 Multi-molecule calculations with CO_2

985 Melamine

986	C	0	3.41626500	-1.58057300	-0.08179400
987	N	0	4.71843000	-1.84590400	0.04346200
988	C	0	5.49499400	-0.74918800	-0.00543100
989	N	0	5.08081300	0.51543000	-0.16282500
990	C	0	3.75263700	0.62380700	-0.30252000
991	N	0	2.85666400	-0.37217800	-0.26592600
992	N	0	2.56157700	-2.63262000	0.02222900
993	N	0	6.82909300	-0.96800600	0.09840800
994	N	0	3.26656300	1.87179600	-0.53421100
995	H	0	2.96058800	-3.55241900	-0.06154500
996	H	0	1.61314800	-2.48952800	-0.28569300
997	H	0	7.07655000	-1.90862200	0.36846900
998	H	0	3.88460300	2.64212200	-0.34193200

999	H	0	2.27525400	2.01062000	-0.41908200
1000	C	0	7.80650800	0.08625600	0.27493400
1001	C	0	9.20211200	-0.44514900	0.00713000
1002	C	0	10.26698100	0.61627300	0.14461700
1003	H	0	7.55716700	0.89481400	-0.41883800
1004	H	0	7.75318600	0.51668000	1.28296500
1005	H	0	9.26295800	-0.86525500	-1.00080800
1006	H	0	9.45281000	-1.25112400	0.70766000
1007	O	-1	10.17902900	1.12348100	1.45823000
1008	H	0	10.09997300	1.43210300	-0.58076700
1009	O	-1	11.52082200	0.01575500	-0.11137900
1010	H	-1	10.87567100	1.77622800	1.57982500
1011	H	-1	12.11084800	0.67628900	-0.48438500
1012	C	0	-0.00303600	0.03981400	-0.52145400
1013	O	0	-0.16387900	-1.11068900	-0.53783100
1014	O	0	0.15789300	1.19028200	-0.51745500
1015	C	0	-3.43873600	1.62912600	-0.07211400
1016	N	0	-4.74361600	1.87695400	0.06271100
1017	C	0	-5.50668600	0.77133500	0.00388700
1018	N	0	-5.07734600	-0.48574800	-0.17177200
1019	C	0	-3.74890000	-0.57619400	-0.31977400
1020	N	0	-2.86518600	0.43028900	-0.27448300

1021	N	0	-2.59627000	2.68991700	0.04154200
1022	N	0	-6.84358700	0.97021400	0.11723700
1023	N	0	-3.24929200	-1.81513400	-0.57031900
1024	H	0	-3.00593100	3.60627400	-0.02667200
1025	H	0	-1.64663100	2.56225400	-0.26944300
1026	H	0	-7.10267600	1.90245700	0.40498700
1027	H	0	-3.85755800	-2.59484000	-0.38474500
1028	H	0	-2.25590100	-1.94390700	-0.46130100
1029	C	0	-7.79990300	-0.10361400	0.29235500
1030	C	0	-9.20877200	0.39223600	0.02483700
1031	C	0	-10.23609200	-0.70813200	0.13977500
1032	H	0	-7.53222100	-0.90510400	-0.40257500
1033	H	0	-7.73692400	-0.53521700	1.29935100
1034	H	0	-9.27706200	0.82410200	-0.97762300
1035	H	0	-9.48866100	1.17950200	0.73538300
1036	O	-1	-10.14023400	-1.22951700	1.44747300
1037	H	0	-10.03061600	-1.50732700	-0.59425100
1038	O	-1	-11.51187500	-0.15653100	-0.12045300
1039	H	-1	-10.80675700	-1.91652900	1.54714800
1040	H	-1	-12.06498600	-0.83687300	-0.51385700
1041	AA-Arg				
1042	C	0	10.01388100	-0.67027400	-0.54729800

1043	O	-1	9.02590800	-1.26670200	-1.33368200
1044	O	-1	10.28618700	-1.50752500	0.55166600
1045	H	-1	10.70768300	-0.92611800	1.20359700
1046	C	0	9.52903800	0.70762100	-0.08250300
1047	H	-1	9.31852300	-2.16983700	-1.49797800
1048	H	0	10.94234400	-0.54060400	-1.13998500
1049	C	0	8.18900500	0.63717600	0.64654500
1050	C	0	6.98708300	0.73764500	-0.28057800
1051	C	0	5.67468900	0.51929000	0.44598000
1052	N	0	4.56618500	0.60425200	-0.48645200
1053	H	0	8.15037400	-0.29456700	1.22316700
1054	H	0	7.08509600	-0.00550100	-1.07823400
1055	H	0	5.53777000	1.28047700	1.22422500
1056	C	0	3.24315100	0.41672500	-0.11818300
1057	N	0	10.57387200	1.18468900	0.83438400
1058	H	0	10.25942900	2.01610700	1.32258500
1059	H	0	11.40392500	1.45812500	0.31555400
1060	H	0	9.42087000	1.34376200	-0.97678200
1061	H	0	8.14367600	1.45006400	1.38349300
1062	H	0	6.96870700	1.72029300	-0.76850100
1063	H	0	5.70948400	-0.45837200	0.95971100
1064	H	0	4.77396200	0.26416600	-1.41420100

1065	N	0	3.00792700	0.50043600	1.24398400
1066	N	0	2.24876100	0.26506800	-0.92321300
1067	H	0	2.55824800	0.34255500	-1.89017800
1068	H	0	2.06551300	0.20327700	1.46272400
1069	H	0	3.69120800	0.04586200	1.83338100
1070	C	0	0.03144900	-1.25558300	-0.07869200
1071	O	0	-0.41999200	-1.30354100	-1.14877200
1072	O	0	0.48601000	-1.30220100	0.98999700
1073	C	0	-10.03656700	-0.60003800	0.67816700
1074	O	-1	-9.04693700	-1.21689800	1.44748200
1075	O	-1	-10.38305300	-1.45550300	-0.38520800
1076	H	-1	-10.79806100	-0.87524800	-1.04231300
1077	C	0	-9.51218600	0.74252000	0.15694500
1078	H	-1	-9.37772000	-2.09828800	1.65246900
1079	H	0	-10.93679400	-0.41452000	1.29886900
1080	C	0	-8.19896500	0.59791100	-0.60903500
1081	C	0	-6.96744600	0.68364600	0.27955200
1082	C	0	-5.68178700	0.42272300	-0.48021300
1083	N	0	-4.54642600	0.50225900	0.41933200
1084	H	0	-8.21162400	-0.35196900	-1.15643400
1085	H	0	-7.05976800	-0.04247900	1.09343400
1086	H	0	-5.55107600	1.16718300	-1.27535200

1087	C	0	-3.23435100	0.31083300	0.01752800
1088	N	0	-10.56540500	1.23541500	-0.74190800
1089	H	0	-10.23332300	2.03908300	-1.26374900
1090	H	0	-11.36709900	1.55739700	-0.20658600
1091	H	0	-9.35109100	1.39942500	1.02792100
1092	H	0	-8.14647700	1.38559800	-1.37247400
1093	H	0	-6.91086300	1.67389800	0.74886300
1094	H	0	-5.75110500	-0.56287400	-0.97496100
1095	H	0	-4.73026300	0.17527700	1.35669100
1096	N	0	-3.03589600	0.37755100	-1.35136100
1097	N	0	-2.21869600	0.16986500	0.79768000
1098	H	0	-2.50199700	0.26079500	1.77146800
1099	H	0	-2.09688700	0.08614500	-1.59193700
1100	H	0	-3.73185500	-0.08807000	-1.91676900
1101	7-Azaindole				
1102	H	0	2.90499600	-2.51720400	-0.48093300
1103	N	0	3.18051300	1.90935800	-0.40838400
1104	C	0	4.26519300	2.75402500	-0.36819900
1105	C	0	5.42313900	2.02144500	-0.34071200
1106	C	0	5.04120300	0.64579100	-0.36727100
1107	C	0	3.62512000	0.61206000	-0.41017400
1108	C	0	5.71617500	-0.58477600	-0.36581600

1109	C	0	4.91232100	-1.72082400	-0.40817400
1110	C	0	3.51560100	-1.61690600	-0.44762300
1111	N	0	2.84465900	-0.46413300	-0.44791000
1112	H	0	2.20550100	2.16703100	-0.43829300
1113	H	0	4.12194800	3.82381800	-0.36561400
1114	H	0	6.42422100	2.42661000	-0.31872500
1115	H	0	5.37028600	-2.70552500	-0.41282800
1116	C	0	7.20857700	-0.65171800	-0.27179800
1117	C	0	7.69320200	-0.40092100	1.15746500
1118	C	0	9.19562000	-0.22968100	1.25315600
1119	H	0	7.57150500	-1.62553600	-0.60946400
1120	H	0	7.66174100	0.09916400	-0.92875700
1121	H	0	7.39003000	-1.22652400	1.81089400
1122	H	0	7.22067200	0.50143200	1.56341300
1123	O	-1	9.79940800	-1.36755300	0.71964500
1124	H	0	9.49088200	-0.08672200	2.30937600
1125	O	-1	9.66595000	0.89040600	0.50102300
1126	H	-1	10.74156500	-1.17793100	0.65335200
1127	H	-1	9.54589900	1.67625500	1.04353000
1128	C	0	0.00240800	-0.00879100	-0.46281200
1129	O	0	0.22298800	1.13187600	-0.46780500
1130	O	0	-0.21780900	-1.14950000	-0.45953200

1131	H	0	-2.88153900	2.50482100	-0.39240300
1132	N	0	-3.18056300	-1.92052600	-0.35029800
1133	C	0	-4.27001300	-2.75987000	-0.33211800
1134	C	0	-5.42448100	-2.02158700	-0.31397400
1135	C	0	-5.03531800	-0.64771000	-0.32461000
1136	C	0	-3.61857700	-0.62097600	-0.34855000
1137	C	0	-5.70396000	0.58632500	-0.32602300
1138	C	0	-4.89370800	1.71842500	-0.35011600
1139	C	0	-3.49723200	1.60761700	-0.37150000
1140	N	0	-2.83232100	0.45134600	-0.37027600
1141	H	0	-2.20688600	-2.18344300	-0.37780900
1142	H	0	-4.13221900	-3.83037500	-0.33623100
1143	H	0	-6.42776200	-2.42177300	-0.30805800
1144	H	0	-5.34665500	2.70543100	-0.35648500
1145	C	0	-7.19745400	0.66132300	-0.26127500
1146	C	0	-7.71777000	0.40444100	1.15431700
1147	C	0	-9.22380500	0.24218200	1.20788500
1148	H	0	-7.54713000	1.63939900	-0.60048600
1149	H	0	-7.64045000	-0.08232400	-0.93325200
1150	H	0	-7.42650000	1.22418600	1.82037400
1151	H	0	-7.26181400	-0.50362200	1.56634900
1152	O	-1	-9.80254900	1.38743300	0.66238300

1153	H	0	-9.55061700	0.09611100	2.25427800
1154	O	-1	-9.67778200	-0.87027900	0.43618400
1155	H	-1	-10.74195500	1.20646700	0.54933300
1156	H	-1	-9.59081700	-1.66034900	0.97886700
1157	TBD				
1158	H	0	-3.23897000	1.40822900	-1.64888800
1159	N	0	-2.88698900	-2.25898800	0.28346900
1160	H	0	-1.88100300	-2.16837700	0.21798800
1161	C	0	-3.43328000	-3.53454900	-0.13179100
1162	H	0	-2.93726700	-4.32603700	0.43695300
1163	C	0	-4.92060600	-3.51822000	0.13221400
1164	H	0	-5.10092900	-3.46002700	1.21038200
1165	C	0	-5.54025400	-2.31711900	-0.55025100
1166	H	0	-6.59471800	-2.25146600	-0.27997700
1167	N	0	-4.87843300	-1.07356300	-0.15955400
1168	C	0	-3.48223400	-1.06335000	-0.09883100
1169	N	0	-2.70009500	-0.05295400	-0.25861200
1170	C	0	-3.28914500	1.22863500	-0.56285400
1171	H	0	-5.16517900	2.28399100	-0.45651000
1172	C	0	-4.72236000	1.35263000	-0.09298000
1173	C	0	-5.55085600	0.16995600	-0.56972700
1174	H	0	-3.24676500	-3.74268900	-1.20038700

1175	H	0	-5.39222600	-4.43294800	-0.23656600
1176	H	0	-5.50492900	-2.44376100	-1.64779700
1177	H	0	-2.67001500	2.01182400	-0.11032700
1178	H	0	-4.75575700	1.38292700	1.00448000
1179	H	0	-5.61365800	0.20579000	-1.67496900
1180	C	0	-6.97312600	0.21892600	-0.00689400
1181	C	0	-7.70122900	1.52672800	-0.27764800
1182	C	0	-9.16588000	1.49790200	0.14897100
1183	H	0	-7.57144800	-0.58642300	-0.44412800
1184	H	0	-6.92651600	0.02304400	1.07169400
1185	H	0	-7.66854000	1.76653600	-1.34744500
1186	H	0	-7.21422600	2.36435500	0.23704400
1187	O	-1	-9.90747500	0.50637100	-0.49810200
1188	H	0	-9.65042800	2.44786300	-0.12109400
1189	O	-1	-9.29456300	1.25393400	1.54540500
1190	H	-1	-8.66926300	1.82287000	2.00523500
1191	H	-1	-9.73129900	-0.30965100	-0.01665400
1192	C	0	-0.01136800	0.01946000	0.57839700
1193	O	0	0.03576600	-1.14127000	0.62150100
1194	O	0	-0.06011400	1.18099400	0.58447000
1195	H	0	3.20604500	-1.47068100	-1.50248900
1196	N	0	2.88836000	2.27676800	0.29376800

1197	H	0	1.88238000	2.18705600	0.22690000
1198	C	0	3.44187400	3.53628700	-0.15846600
1199	H	0	2.94223800	4.34743800	0.37843900
1200	C	0	4.92575500	3.52313800	0.12352000
1201	H	0	5.09206600	3.49522500	1.20514600
1202	C	0	5.55083800	2.30202100	-0.51708400
1203	H	0	6.59978500	2.23913400	-0.22485600
1204	N	0	4.87750300	1.07017300	-0.10901000
1205	C	0	3.48115000	1.06788600	-0.04800200
1206	N	0	2.69565300	0.05543800	-0.17431400
1207	C	0	3.27596700	-1.24098900	-0.42710700
1208	H	0	5.15189300	-2.29446700	-0.31685400
1209	C	0	4.71693700	-1.35059400	0.02296700
1210	C	0	5.53525900	-0.18466100	-0.50925800
1211	H	0	3.26768000	3.70946300	-1.23534700
1212	H	0	5.40569400	4.42531900	-0.26487600
1213	H	0	5.53656400	2.40160900	-1.61798900
1214	H	0	2.66160700	-1.99991900	0.07119800
1215	H	0	4.77090600	-1.34315400	1.11993400
1216	H	0	5.56140900	-0.24864700	-1.61480200
1217	C	0	6.97592400	-0.22397100	0.00467500
1218	C	0	7.68123700	-1.54969700	-0.23807100

C	0	9.16679600	-1.51334300	0.10725100
H	0	7.56415500	0.55673600	-0.48777900
H	0	6.97122800	0.01685900	1.07521200
H	0	7.59055000	-1.84207300	-1.29132300
H	0	7.21659300	-2.35632900	0.34254600
O	-1	9.88081700	-0.57059000	-0.63646700
H	0	9.62709400	-2.48329900	-0.13276600
O	-1	9.37445800	-1.19076900	1.47762300
H	-1	8.76729700	-1.72059200	2.00371900
H	-1	9.74645300	0.27434200	-0.19289500

Multi-molecule calculations with H_2O

Melamine

C	0	3.26009500	-1.14544400	-0.48357200
N	0	4.54200000	-1.39891100	-0.77529800
C	0	5.41172000	-0.57456400	-0.16915200
N	0	5.10573100	0.44243900	0.64979000
C	0	3.78886000	0.58107400	0.85192700
N	0	2.80749300	-0.16585300	0.33063800
N	0	2.32818500	-1.94612000	-1.04024100
N	0	6.72783400	-0.81860000	-0.40864200
N	0	3.41029500	1.62398600	1.64595300
H	0	2.63291400	-2.62671700	-1.71344200

1241	H	0	1.34447500	-1.72750500	-0.93074500
1242	H	0	6.89412000	-1.49735900	-1.13703600
1243	H	0	4.13170500	2.01601500	2.22932900
1244	H	0	2.48175800	1.58385400	2.03343800
1245	C	0	7.79893600	0.09024900	-0.04549500
1246	C	0	9.15892800	-0.54941700	-0.28771300
1247	C	0	10.28901100	0.44512600	-0.13422600
1248	H	0	7.71987100	1.02988800	-0.61546400
1249	H	0	7.68592400	0.36648400	1.00444600
1250	H	0	9.21630600	-0.95999200	-1.30199600
1251	H	0	9.32913100	-1.38011100	0.40490600
1252	O	-1	10.21427400	0.95935600	1.17603100
1253	H	0	10.16834800	1.26763300	-0.86173900
1254	O	-1	11.52967900	-0.20109300	-0.37765300
1255	H	-1	10.91820900	1.60824800	1.27399000
1256	H	-1	12.12217100	0.44387500	-0.77329300
1257	C	0	-3.45030300	1.59743500	-0.73198700
1258	N	0	-4.77120800	1.77095400	-0.73439800
1259	C	0	-5.45396700	0.71215500	-0.25430700
1260	N	0	-4.93086600	-0.43561800	0.19388600
1261	C	0	-3.58893300	-0.47240200	0.13294300
1262	N	0	-2.78303700	0.50748600	-0.32038700

1263	N	0	-2.69327000	2.64949800	-1.15916400
1264	N	0	-6.80113500	0.85351600	-0.23867300
1265	N	0	-2.98380800	-1.60724800	0.54382500
1266	H	0	-3.17910100	3.35474400	-1.68912300
1267	H	0	-1.74681200	2.44432600	-1.43503200
1268	H	0	-7.13712700	1.72018300	-0.62954200
1269	H	0	-3.54708200	-2.30004500	1.00510800
1270	H	0	-1.97548700	-1.62592000	0.63350600
1271	C	0	-7.75378200	-0.17750500	0.12547700
1272	C	0	-9.16986900	0.33894100	-0.08587500
1273	C	0	-10.22980200	-0.72338700	0.10101600
1274	H	0	-7.57844800	-1.07711500	-0.48110900
1275	H	0	-7.60533300	-0.47936200	1.16701400
1276	H	0	-9.27765900	0.73446700	-1.10150200
1277	H	0	-9.38947800	1.16084200	0.60481300
1278	O	-1	-10.12541100	-1.19566400	1.42461000
1279	H	0	-10.06372700	-1.55607600	-0.60527200
1280	O	-1	-11.50256200	-0.14988400	-0.15684300
1281	H	-1	-10.79741400	-1.87337300	1.54841000
1282	H	-1	-12.06597000	-0.83464700	-0.52725100
1283	O	0	-0.13936000	-0.72951900	0.03386700
1284	H	0	0.64909300	-0.25859500	0.35153600

1285	H	0	-0.81621300	-0.06156600	-0.17928200
1286	AA-Arg				
1287	C	0	10.41445900	0.60402200	0.54946500
1288	O	-1	9.54199000	1.19822700	1.44865900
1289	O	-1	10.80152600	1.54728100	-0.42017100
1290	H	-1	11.22170300	1.00445900	-1.10538900
1291	C	0	9.68785800	-0.58921900	-0.09091900
1292	H	-1	9.83608000	2.08975500	1.66540100
1293	H	0	11.32035000	0.23436700	1.07361100
1294	C	0	8.33322700	-0.19923100	-0.69603100
1295	C	0	7.16245500	-0.41059700	0.25928200
1296	C	0	5.80556400	-0.07385200	-0.34237800
1297	N	0	4.75131300	-0.36600600	0.62547700
1298	H	0	8.37669000	0.84416600	-1.02848300
1299	H	0	7.31736500	0.18117800	1.16629900
1300	H	0	5.65104700	-0.62891500	-1.28074400
1301	C	0	3.39597600	-0.34118800	0.32973000
1302	N	0	10.60467600	-1.08054800	-1.13013100
1303	H	0	10.12194100	-1.71660100	-1.75508600
1304	H	0	11.35825100	-1.61742300	-0.71012100
1305	H	0	9.51648800	-1.33379700	0.70448600
1306	H	0	8.16754100	-0.79715600	-1.60217000

1307	H	0	7.15085000	-1.46401300	0.57663300
1308	H	0	5.77885700	0.99164800	-0.60478400
1309	H	0	4.99136200	-1.10199000	1.27451900
1310	N	0	3.03782700	0.48584100	-0.71422600
1311	N	0	2.47960800	-1.01770600	0.94039900
1312	H	0	2.88849300	-1.66795600	1.60702400
1313	H	0	2.03110100	0.63882900	-0.74584500
1314	H	0	3.57195900	1.33858900	-0.79859400
1315	C	0	-10.41118300	0.64279900	-0.54065000
1316	O	-1	-9.52032400	1.34368000	-1.34145900
1317	O	-1	-10.85512900	1.47644100	0.50289700
1318	H	-1	-11.27037000	0.85927300	1.12530100
1319	C	0	-9.68086200	-0.59635300	0.00025700
1320	H	-1	-9.84989100	2.23607200	-1.49420100
1321	H	0	-11.28880100	0.31217600	-1.13414400
1322	C	0	-8.34066900	-0.25190500	0.66286100
1323	C	0	-7.15501700	-0.36862700	-0.29049400
1324	C	0	-5.80586200	-0.08870200	0.35592500
1325	N	0	-4.74202000	-0.28964200	-0.62468400
1326	H	0	-8.39782700	0.75669600	1.08760700
1327	H	0	-7.29994100	0.30568400	-1.13974100
1328	H	0	-5.66065000	-0.72942700	1.23956400

1329	C	0	-3.38948300	-0.29749900	-0.31627800
1330	N	0	-10.60902300	-1.19640200	0.96998400
1331	H	0	-10.12749600	-1.88141100	1.54187600
1332	H	0	-11.34533400	-1.70261800	0.48607200
1333	H	0	-9.48568000	-1.26005200	-0.85872100
1334	H	0	-8.18418200	-0.92889400	1.51328200
1335	H	0	-7.13589600	-1.38739500	-0.70566400
1336	H	0	-5.78293600	0.94764200	0.71702700
1337	H	0	-4.97754700	-0.95786000	-1.34476500
1338	N	0	-3.03865300	0.42262700	0.80632900
1339	N	0	-2.46922000	-0.91374700	-0.98234500
1340	H	0	-2.87317600	-1.49441400	-1.71309000
1341	H	0	-2.03215200	0.56978100	0.86079500
1342	H	0	-3.57287800	1.26361400	0.97042200
1343	O	0	0.00417600	0.22492400	0.04300200
1344	H	0	-0.62280700	-0.33441600	-0.45363200
1345	H	0	0.62924600	-0.38281700	0.48159500
1346	7-Azaindole				
1347	H	0	3.16669800	-2.48743600	-1.83312600
1348	N	0	2.63784300	1.16117300	0.62652000
1349	C	0	3.54417500	2.06544800	1.12151700
1350	C	0	4.81557600	1.69894400	0.75979400

1351	C	0	4.70260900	0.49326500	0.00193800
1352	C	0	3.31485300	0.19395900	-0.06565800
1353	C	0	5.58678300	-0.38750300	-0.64747000
1354	C	0	4.99002000	-1.46665000	-1.29717800
1355	C	0	3.60037400	-1.64185700	-1.30462200
1356	N	0	2.73434900	-0.82590700	-0.69843600
1357	H	0	1.62741700	1.14408000	0.74263400
1358	H	0	3.20824300	2.91314900	1.69910900
1359	H	0	5.71393200	2.24566000	1.00351800
1360	H	0	5.61110800	-2.18669700	-1.82106800
1361	C	0	7.07603700	-0.18369100	-0.65921900
1362	C	0	7.71227500	-0.24050900	0.73021900
1363	C	0	9.22071100	-0.04317100	0.74000900
1364	H	0	7.54569400	-0.93349400	-1.30220200
1365	H	0	7.30845400	0.79035000	-1.10693000
1366	H	0	7.49905200	-1.20177100	1.21024400
1367	H	0	7.26648700	0.52313100	1.37726500
1368	O	-1	9.83153600	-1.17053900	0.18913400
1369	H	0	9.55741700	0.10331100	1.78345900
1370	O	-1	9.65026000	1.08383200	-0.02909700
1371	H	-1	10.76943800	-0.96162800	0.12148200
1372	H	-1	9.51521500	1.86691100	0.51418900

1373	H	0	-3.16572500	2.49494900	-1.80466100
1374	N	0	-2.63516900	-1.16190200	0.64230100
1375	C	0	-3.54107300	-2.06835900	1.13428900
1376	C	0	-4.81272900	-1.70069100	0.77472100
1377	C	0	-4.70044300	-0.49219600	0.02129600
1378	C	0	-3.31275700	-0.19226200	-0.04588600
1379	C	0	-5.58485300	0.38960200	-0.62632400
1380	C	0	-4.98849400	1.47150800	-1.27196200
1381	C	0	-3.59905400	1.64754900	-1.27880700
1382	N	0	-2.73268700	0.82953700	-0.67590900
1383	H	0	-1.62447300	-1.14687500	0.75542800
1384	H	0	-3.20464500	-2.91810200	1.70857500
1385	H	0	-5.71090600	-2.24814800	1.01735900
1386	H	0	-5.60993300	2.19240400	-1.79425100
1387	C	0	-7.07341400	0.18252900	-0.64624300
1388	C	0	-7.72243500	0.22896300	0.73776900
1389	C	0	-9.23217000	0.03875900	0.72372400
1390	H	0	-7.54082900	0.93522400	-1.28747800
1391	H	0	-7.30051700	-0.78903900	-1.10195600
1392	H	0	-7.51098000	1.18506500	1.22871400
1393	H	0	-7.28815600	-0.54294400	1.38278200
1394	O	-1	-9.82379600	1.17361000	0.16734400

1395	H	0	-9.58819300	-0.11125900	1.76015300
1396	O	-1	-9.65211100	-1.08074300	-0.05957700
1397	H	-1	-10.75943900	0.97327500	0.05599500
1398	H	-1	-9.54796700	-1.86887400	0.48312500
1399	O	0	0.00154600	-0.00038800	0.15357400
1400	H	0	0.65727200	-0.43272200	-0.42200600
1401	H	0	-0.66044400	0.43315200	-0.41413000
1402	TBD				
1403	H	0	3.37366600	-1.10733200	-2.27877200
1404	N	0	2.52739000	1.42666900	0.90985300
1405	H	0	1.53707600	1.18666000	0.90438000
1406	C	0	2.86421500	2.82114700	1.08747300
1407	H	0	2.32072600	3.19920100	1.95782000
1408	C	0	4.35848900	2.94294500	1.28018000
1409	H	0	4.64718300	2.47960500	2.22917300
1410	C	0	5.06948100	2.24123200	0.14173500
1411	H	0	6.14503300	2.24520200	0.32280200
1412	N	0	4.62460600	0.85678500	0.01325900
1413	C	0	3.25780100	0.58937000	0.09307000
1414	N	0	2.63619200	-0.41263100	-0.44342800
1415	C	0	3.41402400	-1.36037200	-1.20642100
1416	H	0	5.43166000	-2.09938100	-1.40646800

1417	C	0	4.85827800	-1.43020300	-0.75896700
1418	C	0	5.48211000	-0.04411600	-0.77080500
1419	H	0	2.54992600	3.43662700	0.22583100
1420	H	0	4.66187800	3.99275200	1.31480100
1421	H	0	4.90798200	2.78963500	-0.80379900
1422	H	0	2.94486400	-2.34751700	-1.12184700
1423	H	0	4.91120700	-1.83621000	0.26024300
1424	H	0	5.53107900	0.30506100	-1.81999100
1425	C	0	6.90462000	-0.04197600	-0.19273900
1426	C	0	7.77999100	-1.20001500	-0.65051400
1427	C	0	9.24041400	-1.06611800	-0.22295200
1428	H	0	7.40983700	0.89446800	-0.45451100
1429	H	0	6.82244700	-0.04734400	0.90114600
1430	H	0	7.76628000	-1.29569300	-1.74249400
1431	H	0	7.39462100	-2.14897500	-0.25674800
1432	O	-1	9.92557700	-0.04996200	-0.89468800
1433	H	0	9.78157600	-1.99096000	-0.47222700
1434	O	-1	9.35435400	-0.78079000	1.16684700
1435	H	-1	8.75805300	-1.36855600	1.64129100
1436	H	-1	9.71225100	0.76714900	-0.43041000
1437	H	0	-3.35529400	1.17007500	-2.20378900
1438	N	0	-2.52796400	-1.47437900	0.90815800

1439	H	0	-1.53446600	-1.24840100	0.89626400
1440	C	0	-2.88278400	-2.86795300	1.05473800
1441	H	0	-2.33173700	-3.27645300	1.90633700
1442	C	0	-4.37547000	-2.97106000	1.26814800
1443	H	0	-4.64153900	-2.52622400	2.23246400
1444	C	0	-5.09318800	-2.23177400	0.15820700
1445	H	0	-6.16532800	-2.22261200	0.35832700
1446	N	0	-4.62824500	-0.85162000	0.05208300
1447	C	0	-3.25598800	-0.60682500	0.12151500
1448	N	0	-2.62558700	0.39867200	-0.39761200
1449	C	0	-3.39422300	1.38264200	-1.12274400
1450	H	0	-5.40475600	2.14192800	-1.30484200
1451	C	0	-4.83822000	1.45115800	-0.67432700
1452	C	0	-5.47093500	0.06990900	-0.72426600
1453	H	0	-2.59145800	-3.46543800	0.17261400
1454	H	0	-4.69560100	-4.01635300	1.28263800
1455	H	0	-4.95706800	-2.76134000	-0.80206700
1456	H	0	-2.91487900	2.36113100	-1.00100400
1457	H	0	-4.89099100	1.82940700	0.35546300
1458	H	0	-5.50715900	-0.25669200	-1.78140400
1459	C	0	-6.90218500	0.06441000	-0.16960800
1460	C	0	-7.76252900	1.23323500	-0.62817500

1461	C	0	-9.23766500	1.08821900	-0.26074300
1462	H	0	-7.40658500	-0.86533400	-0.45544600
1463	H	0	-6.84037200	0.05289400	0.92571200
1464	H	0	-7.70960100	1.35546000	-1.71634400
1465	H	0	-7.39306200	2.17272600	-0.19810300
1466	O	-1	-9.89180000	0.08570200	-0.98195900
1467	H	0	-9.76999300	2.01746700	-0.51335300
1468	O	-1	-9.40959800	0.77516700	1.11626700
1469	H	-1	-8.82691500	1.34462500	1.62844800
1470	H	-1	-9.71636300	-0.74202700	-0.52060800
1471	O	0	0.00787100	-0.03694400	0.54154800
1472	H	0	-0.72879300	0.28591900	-0.01626200
1473	H	0	0.74121300	-0.33812400	-0.03241300
1474					
1475					
